# Natural Products–Pyrazine Hybrids: A Review of Developments in Medicinal Chemistry

**DOI:** 10.3390/molecules28217440

**Published:** 2023-11-05

**Authors:** Guo-Qing Chen, Hong-Yan Guo, Zhe-Shan Quan, Qing-Kun Shen, Xiaoting Li, Tian Luan

**Affiliations:** 1 Key Laboratory of Natural Medicines of the Changbai Mountain, Ministry of Education, College of Pharmacy, Yanbian University, Yanji 133002, China; 2022010882@ybu.edu.cn (G.-Q.C.); hongyanguo@ybu.edu.cn (H.-Y.G.); zsquan@ybu.edu.cn (Z.-S.Q.); qkshen@ybu.edu.cn (Q.-K.S.); 2 Department of Pharmacy, Shenyang Medical College, Shenyang 110034, China

**Keywords:** natural products, pyrazine, pharmacological activities

## Abstract

Pyrazine is a six-membered heterocyclic ring containing nitrogen, and many of its derivatives are biologically active compounds. References have been downloaded through Web of Science, PubMed, Science Direct, and SciFinder Scholar. The structure, biological activity, and mechanism of natural product derivatives containing pyrazine fragments reported from 2000 to September 2023 were reviewed. Publications reporting only the chemistry of pyrazine derivatives are beyond the scope of this review and have not been included. The results of research work show that pyrazine-modified natural product derivatives have a wide range of biological activities, including anti-inflammatory, anticancer, antibacterial, antiparasitic, and antioxidant activities. Many of these derivatives exhibit stronger pharmacodynamic activity and less toxicity than their parent compounds. This review has a certain reference value for the development of heterocyclic compounds, especially pyrazine natural product derivatives.

## 1. Introduction

**Figure 1:**
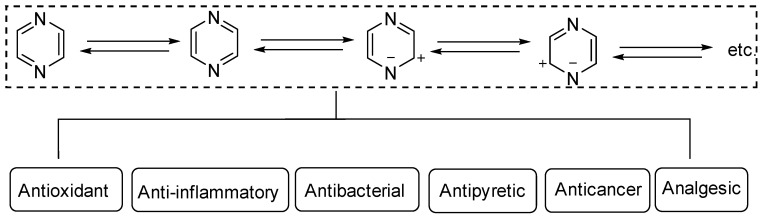
Structure and pharmacological activity of pyrazine.

Heterocyclic structures are common in clinical drugs used to treat diseases. Such drugs typically contain nitrogen, oxygen, and sulfur, which can accept electrons and form hydrogen bonds. These properties enhance the target binding ability of the compound compared to that of hydrocarbons. Heterocyclic compounds are a common class of important compounds in medicinal chemistry and are often used in the synthesis of drugs and other active molecules [1,2,3,4]. Many natural products also contain different kinds of heterocyclic structures. The heterocyclic ring of pyrazine (Figure 1) consists of a six-membered aromatic structure containing two nitrogen atoms, arranged in a 1,4 orientation in the carbon skeleton.

The base of pyrazine (pKa 0.65) is weaker than both pyrazine (pKa 2.3) and pyrimidine (pKa 1.3). Pyrazine can be expressed as a resonance hybrid of some typical structures as shown in Figure 1, which has a resonance energy of 24.3 Kcal/mol and a dipole moment of zero due to the symmetry of the pyrazine molecule. The electron density data show that the electron density of nitrogen atoms increases while that of carbon atoms decreases [5]. Pyrazine is widely used in the synthesis of biologically active ingredients and catalysts, which makes pyrazine a hot topic in pharmaceutical chemistry research. In addition, pyrazine compounds include a variety of pharmacological effects, including antipyretic, anti-inflammatory, analgesic, anticancer, antibacterial, and antioxidant activities [6].

Pyrazine derivatives have been extensively studied as a disorder mediator, and Table 1 shows marketed drugs containing pyrazine structures that have been shown to have biological activity relevant to disease treatment. Many phenazine drugs and compounds containing fragments of pyrazine were also reported that have shown potential therapeutic value, including several that are clinically used to treat human diseases. These results suggest that pyrazine plays an important role in drug discovery [4,7].

Here, we review the pharmacological activities and mechanisms of action of natural products containing pyrazine structures. References are available at Web of Science, PubMed, Science Direct, and SciFinder Scholar. In this paper, the biological activities of natural product derivatives containing pyrazines were reviewed and their mechanism of action was also discussed.

## 2. Natural Product–Pyrazine Hybridization

### 2.1. Acrylic and Cinnamic Acid–Pyrazine Hybridization

**Figure 2:**
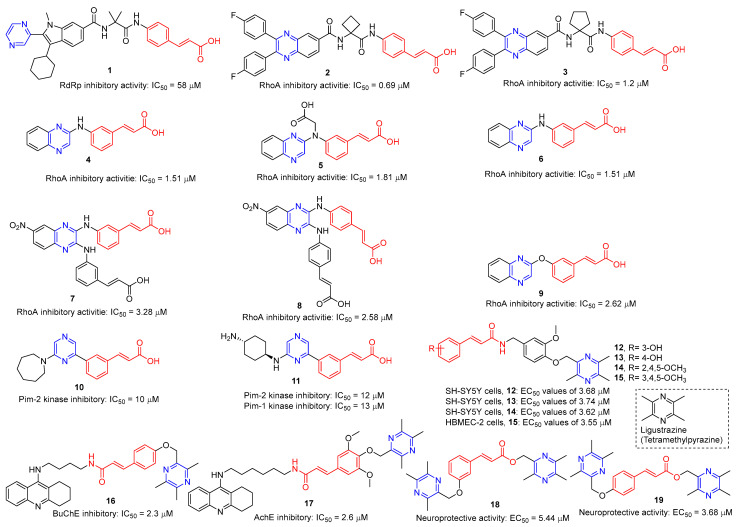
Cinnamic acid–pyrazine derivatives **1**–**19**.

Hepatitis C virus (HCV) is an RNA virus that is spread primarily through contaminated blood. Beaulieu et al. reported the discovery and optimization of specific allosteric inhibitors of NS5B RNA-dependent RNA polymerase (RdRp) encoded by the HCV virus. Derivative **1** (Figure 2) was obtained by introducing pyrazines into C-2 indole substituents. Compound **1** showed good inhibitory activity against RdRp (IC_50_ = 58 μM) and good permeability, solubility, and lipophilicity of caco-2 [69].

Rong et al. identified two cinnamate–pyrazine derivatives **2** and **3** with IC_50_ values of 0.69 and 1.2 μM by using HCV NS5B RdRp for compound library screening [70].

RhoA is a member of Rho GTPase, a subgroup of the Ras superfamily of small GTP-binding proteins. RhoA, as an important regulator of various cell signaling pathways, plays an important role in cytoskeletal organization, transcription, and cell cycle progression. RhoA may be a suitable therapeutic target for the treatment of cardiovascular disease. Ma et al. reported on RhoA inhibitors containing cinnamic acid. Compounds **4** and **5** showed high RhoA inhibitory activity with IC_50_ values of 1.51 and 1.81 μM [71].

Deng et al. reported the RhoA inhibitors of cinnamic acid. Compounds **6**–**9** showed high RhoA inhibitory activity with IC_50_ values of 1.51, 3.28, 2.58, and 2.62 μM. Pharmacological analysis showed that compound **6** had a significant vasodilation effect on PE-induced thoracic aortic ring constriction [72].

A series of Pim-2 kinase inhibitors were identified by Qian et al. through high-throughput screening. Compounds **10** and **11** showed stronger inhibition of Pim-2 kinase with IC_50_ values of 10 and 12 nM. Compound **11** had a stronger inhibitory effect on Pim-1 kinase with an IC_50_ value of 13 nM [73].

Zhang et al. synthesized cinnamic acid–pyrazine derivatives to enhance the bioactivity of cinnamic acid derivatives in neural function and neurovascular protection. The activity of the human microvascular endothelial cell line (HMEC-2) and the human neuroblastoma cell line (SH-SY5Y) against free radical damage increased under the action of compounds **12**–**15**. Compound **15** showed the strongest activity in HBMEC-2 cells with EC_50_ values of 3.55 μM, respectively. Compounds **12**–**14** showed the strongest activity in SH-SY5Y cells, with EC_50_ values of 3.68, 3.74, and 3.62 μM, respectively [74].

Compounds **16** and **17** showed strong inhibitory activity against cholinesterase (ChE). Compound **16** showed the strongest inhibitory effect on BuChE with an IC_50_ of 2.3 nM. Compound **17** had the strongest inhibitory effect on AchE with an IC_50_ of 2.6 nM. Unfortunately, compound **17** had weak inhibition on the self-aggregation of Aβ42 [75].

Wang et al. synthesized a series of ligustrazine–cinnamic acid derivatives as potential neuroprotective agents. Among them, **18** and **19** showed good neuroprotective activity (EC_50_ = 5.44 and 3.68 μM). Compound **19** can inhibit the apoptosis of PC12 cells by blocking the mitochondrial apoptosis pathway by up-regulating the ratio of Bcl-2/Bax, down-regulating the expression of cytochrome-C (Cyt-c), and inhibiting the activities of caspase-9 and caspase-3 [76].

**Figure 3:**
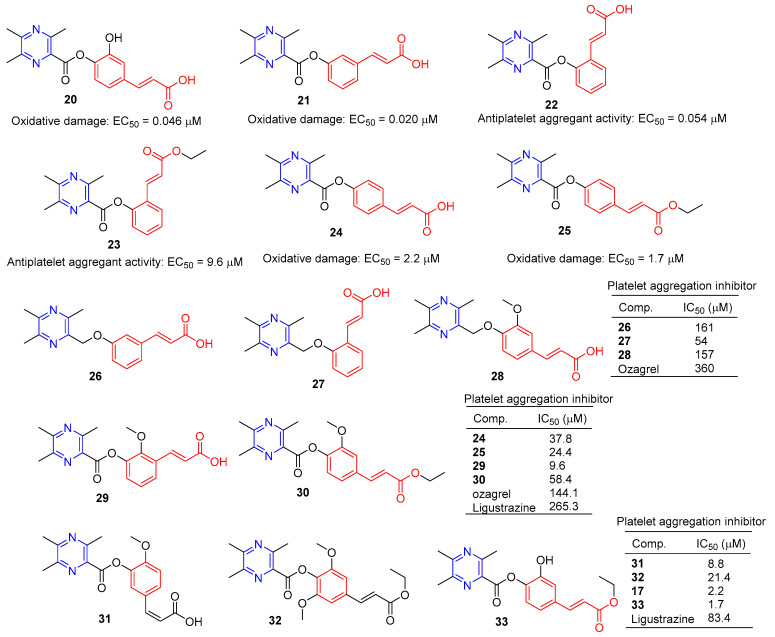
Cinnamic acid–pyrazine derivatives **20**–**33**.

Chen et al. synthesized a series of novel ligustrazine acyloxy cinnamic acid derivatives and studied their in vitro inhibitory effect on adenosine diphosphate (ADP)-induced platelet aggregation and their protective effect on H_2_O_2_-induced oxidative damage of ECV-304 cells. Compounds **20** and **21** (Figure 3) had the highest protective effect on the proliferation of injured ECV-304 cells (EC_50_ = 0.046 and 0.020 μM), and compound **22** had the highest antiplatelet aggregant activity (EC_50_ = 0.054 μM) [77].

Chen et al. evaluated the inhibitory effect of compound **23**–**25** on ADP-induced platelet aggregation in vitro and investigated the protective effect of H_2_O_2_-induced oxidative damage in Ea.hy926 cells. Compounds **24** and **25** showed the highest protective effect on the proliferation of injured Ea.hy926 cells (EC_50_ = 2.2 and 1.7 μM). Compound **23** was the most active antiplatelet aggregator (IC_50_ = 9.6 μM) [78].

Chen et al. synthesized a series of ligustrazine–cinnamic acid derivatives based on the structural characteristics of platelet aggregation inhibitor ozagrel. In particular, compounds **26**–**28** (IC_50_ between 57–161 μM) have a higher platelet aggregation activity than ozagrel (IC_50_ = 360 μM) [77].

To further investigate the antiplatelet aggregation activity of trimethylpyrazine-2-carbonyloxy-cinnamic acids and esters. Chen et al. designed, synthesized, and evaluated a series of new compounds (**24**, **25**, **29**, and **30**); **25** and **29** were the most effective platelet aggregation inhibitors with IC_50_ values of 9.6 and 24.4 μM, respectively, much higher than ozagrel (IC_50_ = 144.1 μM). Chen et al. then tested the protective effect of the compound against hydrogen peroxidation-damaged Ea.hy 926 cells. The corresponding ligustrazine–cinnamic acids/ethyl esters (**17**, **31–33**) had higher activity (IC_50_ = 2.2, 8.8, 21.4, and 1.7 μM) than ligustrazine (IC_50_ = 83.4 μM) [78].

**Figure 4:**
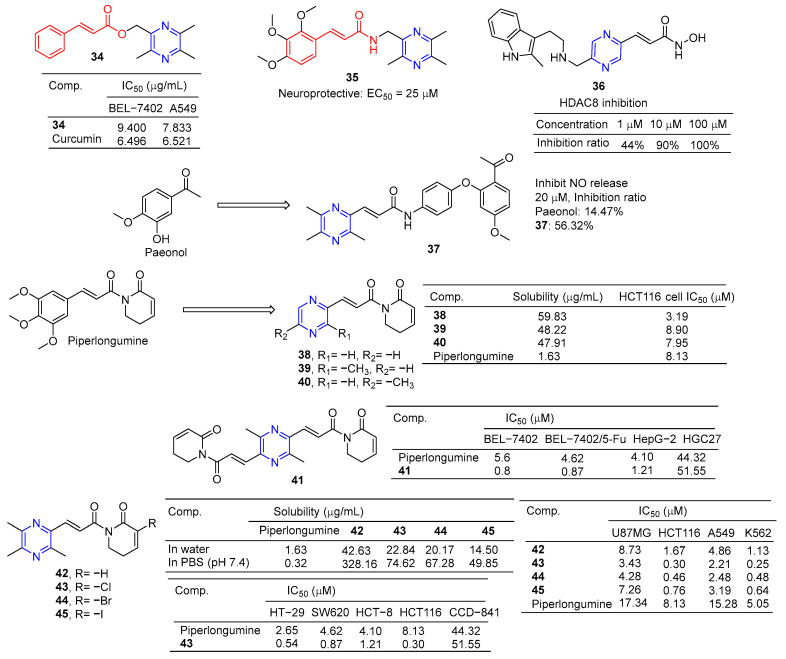
Cinnamic acid–pyrazine derivatives **34**–**45**.

The cinnamic acid–ligustrazine derivative **34** (Figure 4) showed significant inhibitory effects on BEL-7402 and A549 cell lines with IC_50_ values of 9.400 and 7.833 μM [79].

Li et al. reported that ligustrazine–cinnamic acid derivatives showed protective effects against CoCl_2_-induced neurotoxicity in differentiated PC12 cells. The most active compound is **35** (EC_50_ = 25 μM), which exceeds the activity of ligustrazine (EC_50_ = 60 μM) [80].

Balasubramaniam et al. reported the design, synthesis, and evaluation of pyrimidine-based histone deacetylase inhibitors (HDACis). Compound **36** proved to be the most potent inhibitor, producing 100% inhibition at 100 µM, 90% inhibition at 10 µM, and 44% inhibition at 1 µM [81].

Paeonol has been shown to have anti-inflammatory activity, but its anti-inflammatory activity is poor, with only 14.74% inhibitory activity at 20 μM. Hu et al. designed and synthesized a series of paeonol derivatives and screened their anti-inflammatory activities. Compound **37** containing pyrazine structure showed 56.32% inhibitory activity against lipopolysaccharide (LPS)-induced nitric oxide (NO) overexpression in RAW264.7 macrophages at 20 μM [82].

Piperlongumine selectively targets a wide range of cancer cells and induces their death by triggering multiple pathways, including apoptosis, necrosis, and autophagy. Zuo et al. synthesized its analog **38**–**40** by substituting the pyrazine ring for trimethoxyphenyl. These compounds improved water solubility and showed potent anticancer activity against the HCT116 cell line with IC_50_ values of 3.19–8.90 μM [83].

Piperlongumine and ligustrazine have anti-proliferative effects on various types of cancer cells by up-regulating the levels of reactive oxygen species (ROS). Qian et al. designed and synthesized piperlongumine–ligustrazine derivatives and evaluated their bioactivities in vitro and in vivo. Derivative **41** had a more prominent inhibitory effect on the proliferation of drug-sensitive/drug-resistant cancer cells, and the IC_50_ value was lower than that of piperlongumine. The IC_50_ value of **41** pairs of resistant BEL-7402/5-FU cells was 0.9 μM, which was about 9 times higher than that of piperlongumine (IC_50_ = 8.4 μM). Mechanism studies have shown that derivative **41** regulates the DNA damage protein H2AX and autophagy-related proteins LC3, beclin-1, and p62 in drug-resistant BEL-7402/5-FU cells. TrxR activity was inhibited, ROS levels increased, mitochondrial transmembrane potential decreased, and DNA damage and autophagy were dose dependent. Finally, compound **41** showed strong in vivo antitumor activity at 5 mg/kg, with a tumor inhibition rate of 76% (*w*/*w*) [84].

Piperlongumine increases the levels of reactive ROS and induces apoptosis in cancer cells by triggering different pathways. However, the poor solubility of Piperlongumine has limited its further research and clinical application. Ligustrazine has a water-soluble pyrazine skeleton, which can inhibit the proliferation and metastasis of cancer cells. The solubility of compounds **42**–**45** in colorectal cancer HCT116 cells was 8.9–26.2 times higher than that of piperlongumine. Compounds **42**–**45** showed significant inhibitory effects on U87MG, HCT116, A549, and K562 cell lines with IC_50_ values ranging from 0.25 to 8.73 μM. Compound **43** also increased ROS levels. Additionally, compound **43** preferentially inhibited the proliferation, migration, invasion, and heteroadhesion of HCT116 cells. Compound **43** inhibits tumor growth and lung metastasis in vivo and prolongs the survival of tumor-bearing mice. Furthermore, compound **43** mitigated TGF-β1-induced epithelial-mesenchymal transformation and Wnt/βxcatenin activation by inhibiting Akt and GSK-3β phosphorylation in HCT116 cells. Compound **43** has significant anti-proliferation and anti-metastasis activities, which is superior to piperlongumine [85].

### 2.2. Chalcone–Pyrazine Hybridization

**Figure 5:**
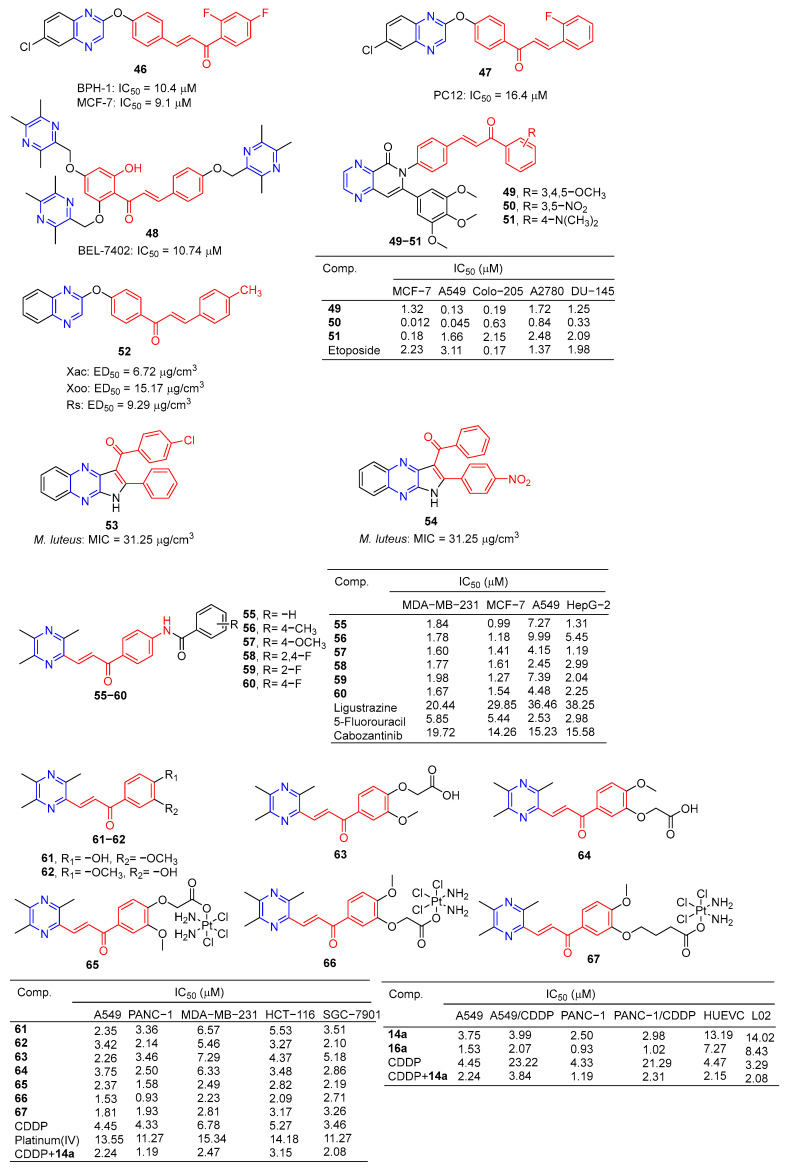
Chalcone–pyrazine derivatives **46**–**67**.

Compound **46** (Figure 5) showed good activity against BPH-1 and MCF-7, with IC_50_ values of 10.4 and 9.1 μM, respectively, comparable to adriamycin (IC_50_ values of 14.1 and 9.2 μM). Compound **47** showed the strongest activity against the PC12 cell line with an IC_50_ value of 16.4 μM [86].

Compound **48** showed the strongest inhibitory effect on the BEL-7402 cell line with an IC_50_ value of 10.74 μM and no toxicity to HUVEC-12 (IC_50_ > 40 μM). Fluorescence staining and flow cytometry analysis showed that compound **48** could induce apoptosis of BEL-7402 cells [87].

Srilaxmi et al. designed and synthesized a series of chalcone–pyazine derivatives and tested the anticarcinogenic activity of all derivatives against five human cancer cell lines (MCF-7, A549, Colo-205, A2780, and DU-145) using a MTT assay. Compound **49** showed significant inhibitory effects on A549 and Colo-205 cell lines with IC_50_ values of 0.13 and 0.19 μM. Compound **50** showed a significant inhibitory effect on the MCF-7 cell line with an IC_50_ value of 0.18 μM. Compound **51** showed significant inhibitory effects on MCF-7, A549, and DU-145 cell lines with IC_50_ values of 0.012, 0.045, and 0.33 μM [88].

The 50% effective concentration (EC_50_) values of compound **52** against *Xanthomonas axonopodis* pv.Citri (Xac), *Xanthomonas oryzae* pv.oryzae (Xoo), and *Ralstonia solanacearum* (Rs) were 6.72, 15.17, and 9.29 μg/cm^3^, respectively, which were better than those of *Bismerthiazol* (44.31, 42.46, and 62.36 μg/cm^3^, respectively) [89].

Compounds **53** and **54** showed good antibacterial activity against *M. luteus*, with a MIC value of 31.25 µg/mL, similar to that of tetracycline (MIC = 31.25 µg/mL) [90].

Luo et al. synthesized a series of ligustrazine–chalcone hybrids and evaluated their antitumor activity in vitro and in vivo. Compounds **55**–**60** showed significant cytotoxicity to MDA-MB-231, MCF-7, A549, and HepG-2 cell lines in vitro, with IC_50_ values ranging from 0.99 to 9.99 μM. Compounds **57** and **60** showed significant effects on the MDA-MB-231 cell line (IC_50_: **57**, 1.60 μM; **60**, 1.67 μM) and MCF-7 cell line (IC_50_: **57**, 1.41 μM; **60**, 1.54 μM) had a good anti-proliferation effect. Compounds **57** and **60** showed strong inhibition of colony formation in both MDA-MB-231 and MCF-7 cell lines, and also showed strong inhibition of migration of these two cell lines in wound healing tests. It should be noted that compound **57** can significantly induce apoptosis of MDA-MB-231 cells in a concentration-dependent manner, inhibit the transformation of the MDA-MB-231 cell growth cycle, and block the cell growth cycle in the G0/G1 phase. Furthermore, compound **57** showed significant antitumor growth efficacy in in vivo anti-proliferation experiments in the NBC model, with a wide safety window. Immunohistochemical analysis showed that compound **57** could significantly reduce the positive rate of Ki-67 in a dose-dependent manner [91].

Wang et al. designed and synthesized six series of ligustrazine–chalketone-modified platinum (IV) complexes and evaluated their anti-proliferation activities. Compounds **61**–**67** showed significant inhibitory effects on A549, PANC-1, MDA-MB-231, HCT116, and SGC-7901 cell lines, with an IC_50_ ranging from 0.93 to 7.29 μM. Among them, **66** showed higher cytotoxicity to cancer cell lines than the cisplatin (CDDP) or combination group, and lower cytotoxicity to normal human cells than the CDDP or combination group. Mechanism studies have shown that **66** effectively induces DNA damage and initiates mitochondrial-dependent apoptosis pathways. In addition, **66** regulates the expression level of nuclear factor erythroid 2-related factor 2, glutathione peroxidase 4, and solute carrier family 7 member 11 expression level, significantly induced iron sag. Furthermore, in pancreatic cancer anti-CDDP xenotransplantation models, **66** achieved better antitumor efficiency in vivo than CDDP, but without significant side effects [92].

### 2.3. Polyphenols–Pyrazine Hybridization

**Figure 6:**
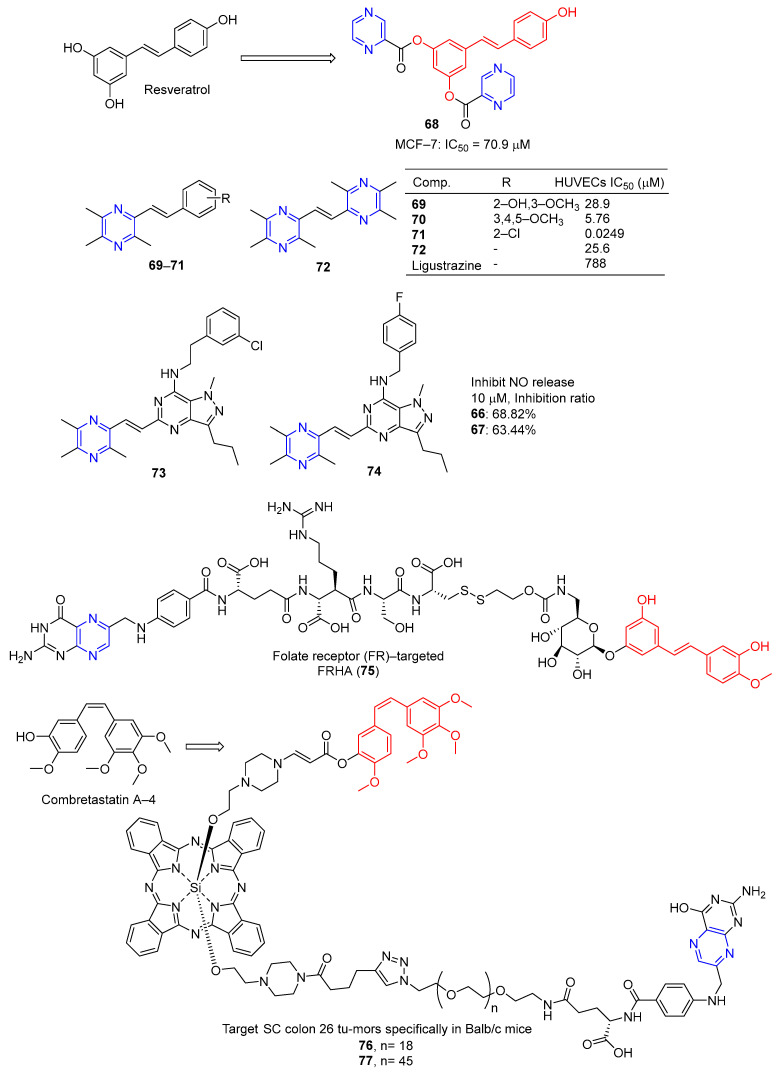
Pyrazine-based resveratrol derivatives, hetero analogs **68**–**75**, and combretastatin A-4-pyrazine derivative **76**–**77**.

Du et al. designed and synthesized a series of heterocyclic analogs of resveratrol and evaluated their inhibitory effects on MCF-7 cells. Among them, compound **67** (Figure 6) linked to pyrazine showed a certain inhibitory effect on MCF-7 with an IC_50_ value of 70.9 μM. The activity exceeded that of resveratrol (IC_50_ = 80.0 μM) [93].

Resveratrol is widely used as a vasodilator, free radical scavenger, and antioxidant, as well as an anti-platelet aggregator and anti-atherosclerotic agent for the prevention and treatment of cardiovascular diseases and ischemia [94]. Deng et al. designed and synthesized a series of ligustrazine–stilbene hybrid derivatives. Derivatives **69**–**72** showed high protective effects on human umbilical cord vascular endothelial cells (HUVECs) damaged by hydrogen peroxide, with values of EC_50_ ranging from 0.0249 to 28.9 μM. Among them, the EC_50_ value of compound **71** is 0.0249 μM, which is 30,000 times higher than that of tetramethylpyrazine (EC_50_ = 788 μM) [95].

Chen et al. designed and synthesized a series of pyrazole–pyrimidine derivatives, and screened their anti-NO activity and toxicity to normal hepatocytes (L02). Compounds **73** and **74** have low toxicity (against L02: IC_50_ = 786.31 and >1000 μM, respectively) and strong anti-NO release effect (IR = 68.82%, 63.44%, at 10 μM, respectively) [96].

Liang et al. synthesized the folate receptor (FR)–targeted rhaponticin conjugate FRHA (**75**) using a hydrophilic peptide separator linked to folate and a disulfide linker. FRHA (**75**) maintains a high affinity for FR-positive cells and produces specific dose-responsive activity in vitro. Treatment of FRHA (**75**) with a reducing agent shows that the amino reactive derivatives of rhaponticin will be released spontaneously after the reduction of disulfide bonds in the nucleosome. In vivo, FRHA (**75**) has also been shown to have specific activity against FR-positive allograft and xenograft models, and possible therapeutic activity leads to mild to moderate toxicity [97].

Nkepang et al. designed and prepared A series of folate–combretastatin A-4 conjugated prodrugs. Prodrugs **76** and **77**, with longer PEG intervals and greater hydrophilicity, enhance the uptake of colon 26 cells by FR-mediated mechanisms and specifically target SC colon 26 tumors in Balb/c mice [93].

**Figure 7:**
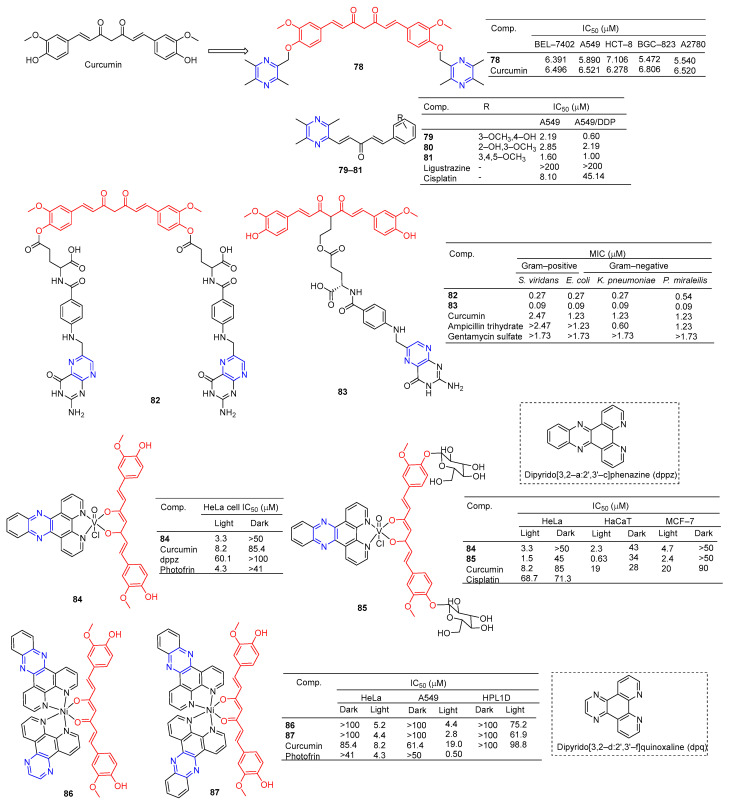
Curcumin–pyrazine derivatives **78**–**87**.

Curcumin is a polyphenolic compound extracted from *Curcuma longa*, which has been extensively studied for its potential anticancer effects [98]. Wang et al. synthesized a series of ligustrazine–curcumin derivatives by coupling antitumor bioactive compounds with ether bonds. Among them, compound **78** (Figure 7) pairs BEL-7402, A549, HCT-8, BGC-823, and A2780 cells cell lines were 6.391, 5.890, 7.106, 5.472, and 5.540 μM [99].

Ai et al. designed and synthesized a series of ligustrazine–curcumin hybrids [71]. Compounds **79**–**81** showed significant inhibitory effects on A549 and A549/DDP cell lines with IC_50_ ranging from 0.60 to 2.85 μM. Pharmacological studies showed that compound **79** inhibited the expression of thioredoxin reductase (TrxR), promoted the accumulation of ROS in cells, and significantly inhibited the apoptosis of proliferation-sensitive (A549, SPCA-1, LTEP-G-2) and drug-resistant (A549/DDP) lung cancer cells. In addition, its antitumor activity was significantly weakened by active oxygen scavenger. In addition, **79** also inhibited NF-κB, AKT, and ERK signaling pathways, P-GP-mediated Rhodamine 123 efflux, P-gp ATPase activity, and P-gp expression in A549/DDP cells [100].

K. Singh et al. synthesized a series of curcumin bioconjugations and tested them for antibacterial and antiviral activity. The antibacterial activity of compounds **82** and **83** against Gram-positive (*S. viridans*) and Gram-negative (*E. coli*, *K. pneumoniae*, and *P. miraleilis*) ranged from 0.09 to 0.54 μM [101].

Curcumin, as a cell imaging and photodynamic therapy (PDT) agent, showed significant photocytotoxicity at visible wavelengths of 400–700 nm with IC_50_ = 8.2 μM. Its degradation is prevented by the formation of phototoxic dipyridophenazine (dppz) complex **84** (IC_50_ = 3.3 μM). However, both compounds are less toxic in the dark (IC_50_ > 50 μM) [102].

Banerjee et al. synthesized a ternary vanadium oxide complex of *O*-phenanthroline with curcumin or disaccharide curcumin, an anticancer compound based on curcumin. Complexes **84** and **85** showed significant phototoxicity at visible light (400–700 nm), with IC_50_ values < 5 μM in HeLa, HaCaT, and MCF-7 cells, and without significant dark toxicity. The DNA ladder, membrane VzFITC/PI, and DCFDA data showed that these complexes lead to apoptotic cell death by forming ROS under light exposure while remaining inert in the dark. Confocal microscopy showed that the complex was mainly located in the cytoplasm, and complex **84** had significant mitochondrial uptake [103].

Banaspati et al. prepared a series of curcumin–nickel (II) complexes and studied their photoinduced anticancer activity in vitro. Curcumin complexes **86** and **87** have REDOX activity in the nickel center, have considerable affinity for binding with calf thymus DNA (ct-DNA), and have moderate affinity for interacting with human serum albumin (HSA). Complexes **86** and **87** exhibit significant photoinduced in vitro cytotoxicity in HeLa and A549 involving reactive ROS with very low dark toxicity. Complexes **86** and **87** are much less toxic to immortalized normal lung epithelial cells (HPL1D). Confocal microscopy images of complexes **86** and **87** show that they are mainly localized in the cytoplasm of A549 cells. JC-1 experiment showed that under visible light irradiation, the sub-G1 cell cycle process of A549 cells was blocked, the mitochondrial membrane potential was significantly lost, and the main mechanism of cell death was apoptosis [104].

### 2.4. Flavono–Pyrazine Hybridization

**Figure 8:**
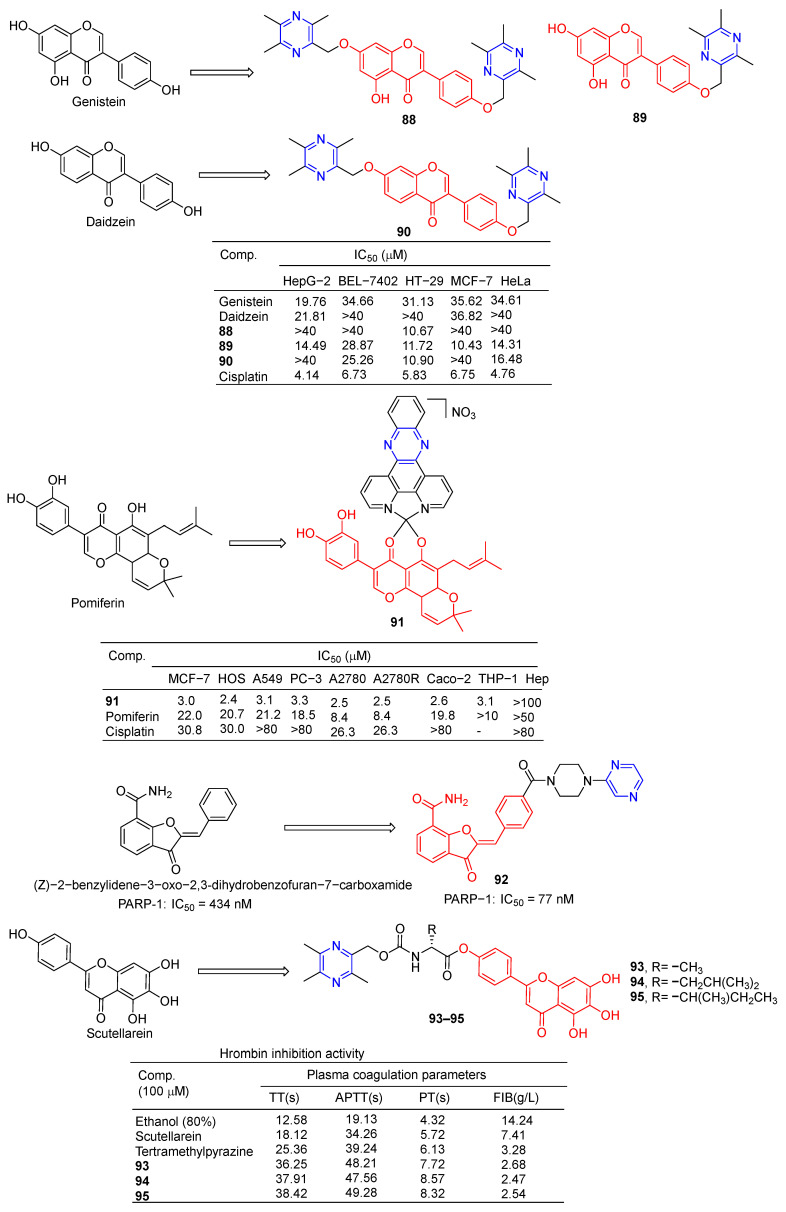
Flavono–pyrazine derivatives **88**–**95**.

Wang et al. designed and synthesized a series of derivatives using ligustrazine and flavonoids as raw materials and tested the antitumor activities of these derivatives. Compounds **88** and **90** (Figure 8) showed the strongest inhibitory effects on HT-29 cell lines, with IC_50_ values of 10.67 and 10.90 μM. Compound **89** showed the strongest inhibitory effect on the MCF-7 cell line with an IC_50_ value of 10.43 μM [87].

Vančo et al. prepared a series of heterologous fish meal containing copper complexes and evaluated their antitumor activity. Complex **91** has significant in vitro cytotoxicity against a variety of human cancer cells (MCF-7, HOS, A549, PC-3, A2780, A2780R, Caco-2, and THP-1) with IC_50_ values of 2.2–3.3 µM. Additionally, complex **91** was less toxic to healthy human hepatocytes, with IC_50_ > 100 µM. Complex **91** is capable of inducing the destruction of intracellular life molecules and subsequent cell death, primarily through the initiation or progression of oxidative stress. Complex **91**, on the other hand, has shown the ability to inhibit inflammation-related signaling pathways (NF-κB/AP-1 activity, NF-κB translocation, and TNF-α secretion) [105].

Poly(ADP-ribose) polymerase (PARP) inhibitors are a class of anticancer drugs that block the catalytic activity of PARP protein. The nerone derivative **92** containing pyrazine showed a significant inhibitory effect on PARP, IC_50_ = 77 nM [106].

Compounds **93**–**95** showed stronger thrombin inhibitory activity than baicalin and TMP, all of which prolonged TT, APTT, and PT to varying degrees, and significantly reduced plasma FIB content at the same concentration. Compounds **93**–**95** showed enhanced neuroprotective and antithrombotic activity against H_2_O_2_-induced PC12 cell death. Compound **93** was used in cerebral ischemia–reperfusion experiments in the middle cerebral artery occlusion (MCAO) model. The results showed that compound **93** could significantly reduce the infarct size of CA1 pyramidal neurons and reduce the damage to neuron cells. Therefore, compound **93** has obvious antioxidant, anticoagulant, and protective effects on brain I/R injury [107].

### 2.5. Coumarin–Pyrazine Hybridization

**Figure 9:**
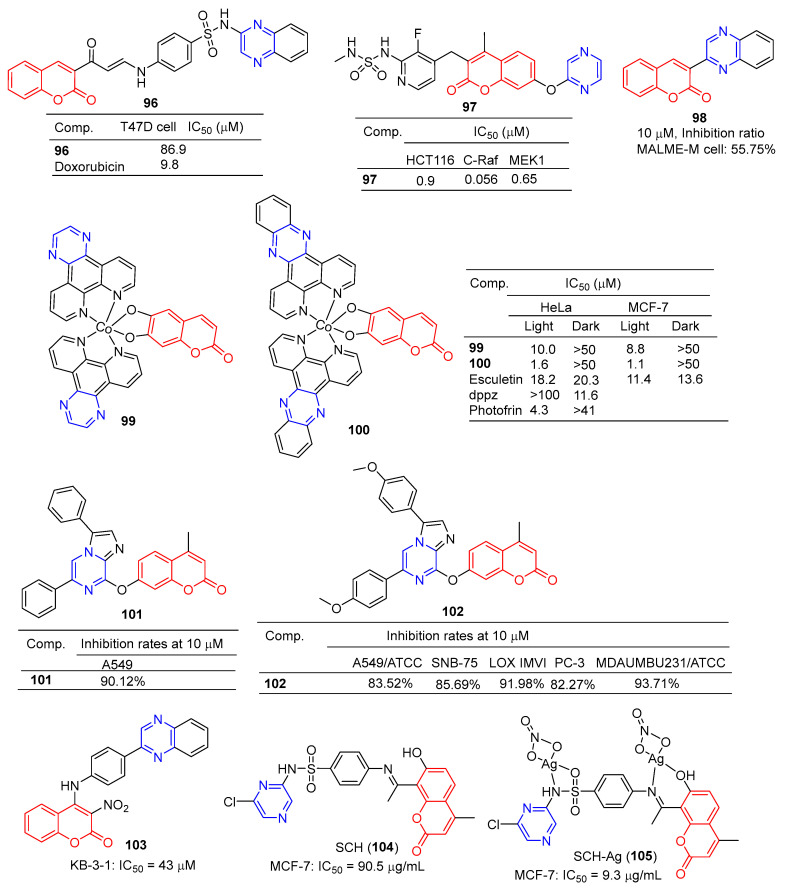
Coumarin–pyrazine derivatives **96**–**105**.

The coumarin derivative **96** (Figure 9) containing sulfonamide showed moderate anticancer activity against the breast cancer cell line (T47D) with an IC_50_ of 86.9 μM [108].

Compound **97** showed significant inhibitory effects in HCT116, C-Raf, and MEK1 cell lines with IC_50_ values of 0.9, 0.056, and 0.65 μM. The effects of the interaction between the derivative **97** and its on-target and off-target proteins (Raf/MEK, CYPs, and hERG channels) were also detected, but the interaction was weaker [109].

The GI inhibition rate of the coumarin derivative **98** in the MALME-M cell line was 55.75% at a concentration of 10 μM [110].

Compounds **99** and **100** showed high visible-light trigger cytotoxicity against HeLa and MCF-7 cancer cells, producing significantly low micromolar IC_50_ values (IC_50_ = 1.1–10.0 μM) and much lower toxicity under dark conditions (IC_50_ > 50 μM). Confocal microscopy showed that compound **100** accumulated in HeLa cells’ mitochondria and induced apoptosis by ROS generation through type 1 photosynthesis [111].

Goel et al. found that arylated imidazo [1,2-α] pyrazine–coumarin hybrids **101** and **102** exhibited significant antitumor activity at a concentration of 10 μM [112].

H. Halawa et al. synthesized a series of new 4-arylamino-3-nitrocoumarin and evaluated the cytotoxic activity of the KB-3-1 cell line in vitro using the resazurin method. Among them, KB-3-1 cells containing the pyrazine derivative **103** showed moderate cytotoxicity with an IC_50_ value of 43 μM [113].

L. El-Ansary et al. prepared a new Schiff base SCH (**104**) using 8-acetyl-7-hydroxy-4-ethylcoumarin and sulfaclozine as raw materials. Its silver complex SCH-Ag (**105**) was also synthesized. The inhibitory effects of SCH (**104**) and SCH-AG (**105**) on a variety of bacteria and fungi and the antitumor activity against MCF-7 cell lines in vitro were evaluated. SCH (**104**) and SCH-AG (**105**) showed strong inhibitory activity against three species of bacteria (*S. aureus*, *B. subtilis*, and *P. aeruginosa*), but no activity against fungi (*A. flavus* and *C. albicans*). Furthermore, the antibacterial activity of SCH-Ag (**105**) was higher than that of SCH (**104**). The IC_50_ of SCH (**104**) for the MCF-7 cell line was 90.5 μg/mL, while that of SCH-Ag (**105**) was 9.3 μg/mL. SCH (**104**) and SCH-Ag (**105**) showed less antitumor activity than cisplatin (IC_50_ = 1.7 μg/mL) [114].

**Figure 10:**
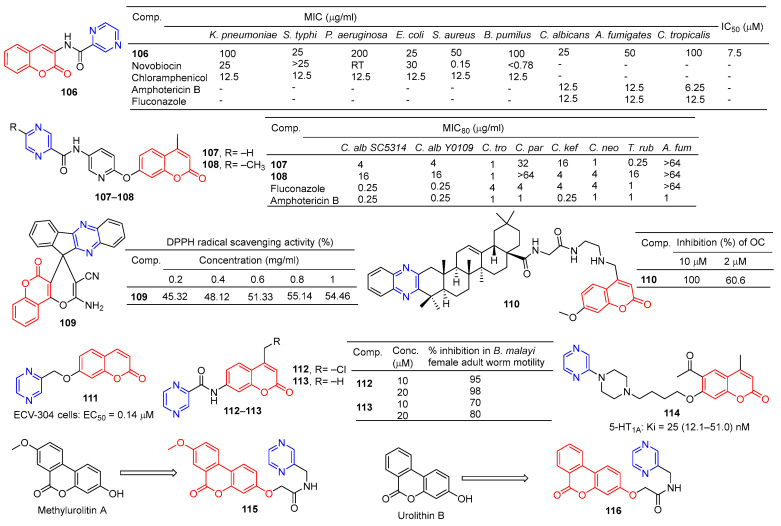
Coumarin–pyrazine derivatives **106**–**116**.

Compound **106** (Figure 10) has good antibacterial activity for strains of *Salmonella typhi* MTCC 537, *Escherichia coli* MTCC 64, and *Candida albicans* MTCC 3017, with a MIC value of 25 µg/mL. Compound **106** showed obvious inhibitory activity against chitinase with an IC_50_ value of 7.5 μM [115].

Chai et al. synthesized a series of 7-*O*-substituted pyridine-4-methyl coumarin derivatives and evaluated their antibacterial activity *in vitro*. Compound **107** showed significant inhibition against *Candida tropicalis*, *Cryptococcus neoformans*, and *Trichophyton rubrum* strains with MIC_80_ values of 1, 1, and 0.25 μg/mL. Compound **108** showed obvious inhibition against *Candida tropicalis* strain with MIC_80_ of 1 μg/mL [116].

Moosavi-Zare et al. synthesized a series of spiro–pyran derivatives and screened their antioxidant activities by a DPPH radical scavenging assay. Spiropyr derivative **109** showed good dose-dependent (0.2–1 mg/mL) free radical resistance (45.32–55.14%) [117].

Compound **110** showed stronger inhibitory activity against RANKL-induced osteoclast differentiation in RAW264.7 cells at 2 μM, with an inhibition rate of 60.6%. Compound **110** showed no cytotoxicity to the RAW264.7 cell line at a concentration of 10 μM [118].

Compound **111** showed significant protective activity against ECV-304 cells (EC_50_ = 0.14 μM), far superior to ligustrazine (EC_50_ = 0.60 μM) [119].

Priyanka et al. synthesized a series of 7-benzamidocoumarin derivatives and evaluated in vitro the antifilarial activity against the human lymphatic filarial parasite, Brugia malayi. There are also pyrazine compounds **112** and **113** with 95% and 70% inhibition of adult motility at 10 μM, which can permanently paralyze the nematode [120].

Ostrowska et al. designed a series of 6-acetyl-7-hydroxy-4-methyl coumarin derivatives containing piperazine groups. Pyrazine-containing derivative **114** showed weak activity against the 5-HT1A receptor with a Ki value of 25 (12.1–51.0) nM [121].

In order to search for potential drugs with good anti-aging effects, Tang et al. synthesized methylurolitin A and its amide derivatives. *Caenorhabditis elegans* (*C. elegans*) was used to evaluate its anti-aging effect and biosafety. Methylurolitin A has good biosecurity for the growth, reproduction, and activity of *C. elegans*. The derivative **115** has the best life-prolonging effect, the anti-aging effect is greater than methylurolitin A, and it has good biosafety [122].

Urolithin B is a natural metabolite that shows good activity in diseases such as obesity, diabetes, osteoporosis, cancer, learning, and memory disorders. Chen et al. designed and synthesized an amide derivative of urolithin B and verified its anti-aging and biosafety using *C. elegans*. The results showed that **116** had the best anti-aging activity among all derivatives, and the compound had good biosafety [123].

### 2.6. Anthraquinone- and 1,4-Naphtoquinone–Pyrazine Hybridization

**Figure 11:**
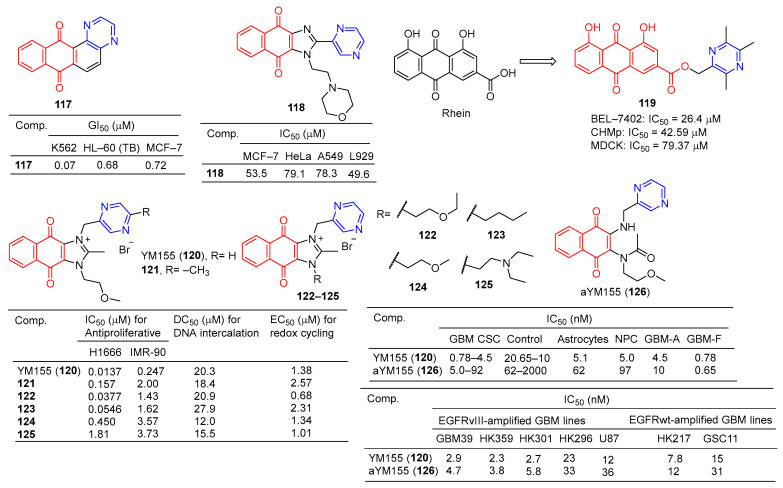
Anthraquinone–pyrazine derivatives **117**–**126**.

The GI_50_ of compound **117** (Figure 11) in leukemia cell lines ranged from 0.07–3.65 μM. The GI_50_ of the breast cancer subgroups ranged from 0.72–19.1 μM. Compound **117** showed the strongest activity against K562 leukemia, with a GI_50_ of 0.07 μM, LC_50_, and TGI of >100 μM, respectively. HL-60 (TB) and MCF-7 cell lines followed, with GI_50_ values of 0.68 and 0.72 μM [124].

Compound **118** showed certain antiproliferative activity against MCF-7, HeLa, and A549 (IC_50_ = 53.5, 79.1, and 78.3 μM, respectively), and high cytotoxicity against L929 (IC_50_ = 49.6 μM) [125].

The derivative of tetramethylpyrazine–rhubaric acid **119** not only inhibited the proliferation of BEL-7402 cancer cells (IC_50_ = 26.4 μM), but also significantly inhibited the normal angiogenesis of the chicken chorionic allantoic bladder [126].

The IC_50_ of derivative **119** for CHMp (canine inflammatory mammary carcinoma cell line) and MDCK (Madin–Darby immortalized canine kidney cell line) is 42.59 μM and 79.37 μM, respectively. Derivative **119** mediates apoptosis through mitochondrial damage, and arrest of the S phase and G2/M phase by down-regulation of cyclin B1. In addition, derivative **119** reduces filamentous foot and inhibits cell migration by downregulating cadherin. In the CMIC lung metastasis model, derivative **119** can effectively inhibit lung tumor growth without obvious toxicity [127].

YM155 (**120**) is a potent broad-spectrum anticancer drug derived from phenotypic screening of inhibitors of survivin expression function. The anticancer drug YM155 (**120**) has been widely studied as a specific statin inhibitor. The IC_50_ value of YM155 (**120**) against the H1299 cell line was 0.0137 μM, and the SI value was 18. Furthermore, YM155 (**120**) has been found to induce DNA damage. Si-Han Sherman Ho et al. synthesized a series of YM155 (**120**)-linked pyrazine derivatives and tested their antitumor activity against H1299 cell lines. Compounds **37** and **38** showed little activity against H1299 cell lines, with IC_50_ values of 0.0377 and 0.0546 μM, but SI values of 38 and 30 were higher than YM155 (**120**) [128].

Iwai et al. found that YM155 (**120**) down-regulates survivin and exhibits strong antitumor activity. In the Caco-2 cell model, YM155 (**120**) was observed as a substrate for P-gp [99]. Premkumar et al. found that YM155 (**120**) at 25 nM down-regulates survivin in gliomas, down-regulates myeloid cell leukemia sequence 1 (Mcl-1), and up-regulates Noxa levels. These findings suggest that YM155 (**120**) negatively regulates Mcl-1 and survivin through endogenous and exogenous apoptotic pathways and amplifies mitochondrial signaling, thus inhibiting glioma cell resistance to TRAIL-induced apoptosis (TRAIL is a tumor necrosis factor-associated apoptosis-inducing ligand). YM155 (**120**) combined with TRAIL significantly increased antitumor activity and may have application value in the treatment of malignant glioma [129].

Ho et al. evaluated the DNA binding affinity of the test compound (**120**–**125**) by monitoring the displacement of thiazole oranges from herring sperm DNA [130,131]. The DC_50_ of YM155 (**120**) was 20.3 μM and that of adriamycin was 2.64 μM. Compounds **121**, **124**, and **125** showed higher activity than YM155 (**120**), with DC_50_ values of 18.4, 12.0, and 15.5 μM, respectively [128].

Glioblastoma (GBM) is the most common primary central nervous system (CNS) malignancy. Furthermore, YM155 (**120**) has clinical tolerance problems due to its lack of cell type selectivity. Thomas J. West et al. synthesized a prodrug of YM155 (**120**), named aYM155 (**121**). aYM155 (**121**) was used against GBM cancer stem-like cells (IC_50_ = 0.7–10 nM) from multiple patient sources. The EGFR variant III-expressing (EGFRvIII) cell line (IC_50_ = 3.8–36 nM) shows strong cell-killing activity and is activated in a cell type-dependent manner. The survivin inhibitory and apoptosis-inducing activity of YM155 (**120**) is related to its interaction with receptor-interacting protein kinase 2 (RIPK2). In an orthotopic intracranial GBM xenograft model, aYM155 (**121**) significantly inhibited brain tumor growth in vivo, which was related to the pharmacodynamics of selective survivin based on cytotypes [132].

**Figure 12:**
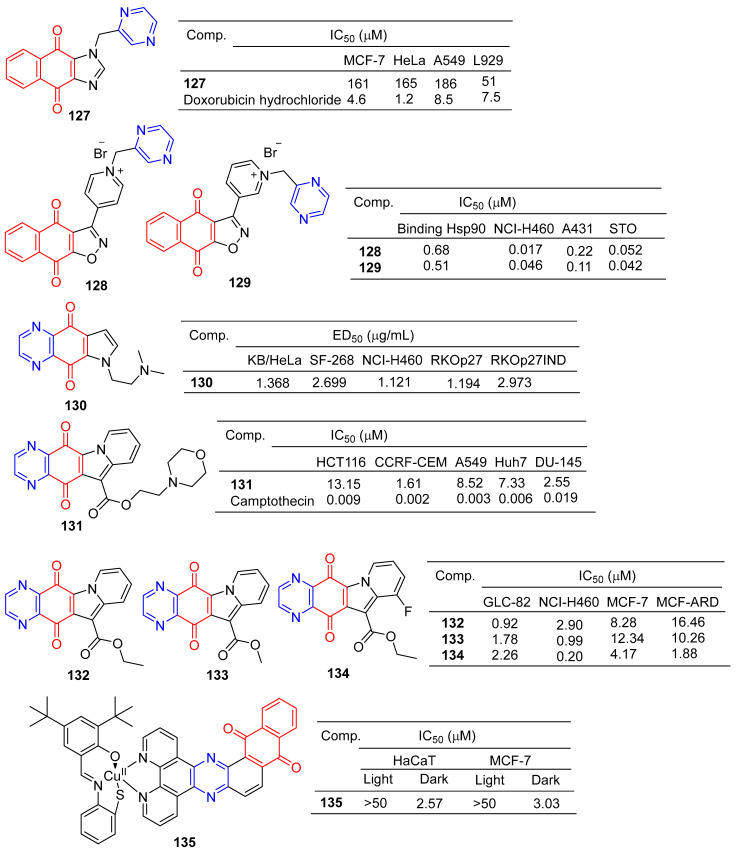
Anthraquinone–pyrazine derivatives **127**–**135**.

Liu et al. designed and synthesized a series of 1-monosubstituted naphthoquinone imidazole derivatives and tested their antitumor activity in vitro. When the substituent was pyrazine, compound **127** (Figure 12) showed a weak inhibitory effect on MCF-7, HeLa, and A549 cell lines, with IC_50_ values ranging from 161–186 μM. However, compound **127** was more toxic to normal cell L929 with IC_50_ of 51 μM [133].

Bargiotti et al. synthesized a series of 3-arylnaphthalene [2,3] isoxazole-4,9-diones and tested the binding of these compounds to Hsp90 and their effects on Hsp90 client proteins expression in human tumor cell lines. The pyrazine-containing compounds **128** and **129** have a strong affinity for Hsp90 with IC_50_ values of 0.68 and 0.51 μM. Additionally, compounds **128** and **129** showed significant inhibitory effects on NCI-H460, A431, and STO cell lines, with IC_50_ values ranging from 0.017 to 0.22 μM [134].

Shanab et al. designed and synthesized a series of azanadione–pyrrolidinated derivatives and evaluated the anti-proliferative activity of all compounds in multiple cell lines. The pyrazine-containing compound **130** showed significant inhibitory activity against KB/HeLa, SF-268, NCI-H460, RKOp27, and RKOp27IND cell lines with IC_50_ values ranging from 1.121–2.973 μM [135].

Yu et al. designed and synthesized derivatives of indolizinoquinolinedione scaffold and tested the antitumor activity of these compounds. The MTT assay showed that compound **131** containing pyrazine showed significant inhibitory effects on HCT116, CCRF-CEM, A549, Huh7, and DU-145 cell lines, with IC_50_ values ranging from 1.61 to 13.15 μM [136].

Shen et al. designed and synthesized a series of new Indolizinoquinoxalin-5,12-dione derivatives. Compounds **132**–**134** showed significant inhibitory effects on the growth of four human tumor cell lines (GLC-82, NCI-H460, MCF-7, and MCF-ARD), with IC_50_ values ranging from 0.20 to 16.46 μM [137].

Devi et al. synthesized a series of new anthraquinone-based copper (II) complexes. Nuclear targeting complex **135** showed significant cytotoxicity to cancer cells in visible light (IC_50_ = 2.57–3.03 µM), but decreased dark toxicity (IC_50_ > 50 µM). Singlet oxygen produced by complex **135** photosensitization is a key cytotoxic substance that causes apoptosis damage in cancer cells. The S-coordination and anthraquinone moiety of complex **135** exhibit double photosensitivity, resulting in a significant PDT effect on cancer cells with minimal dark toxicity [138].

**Figure 13:**
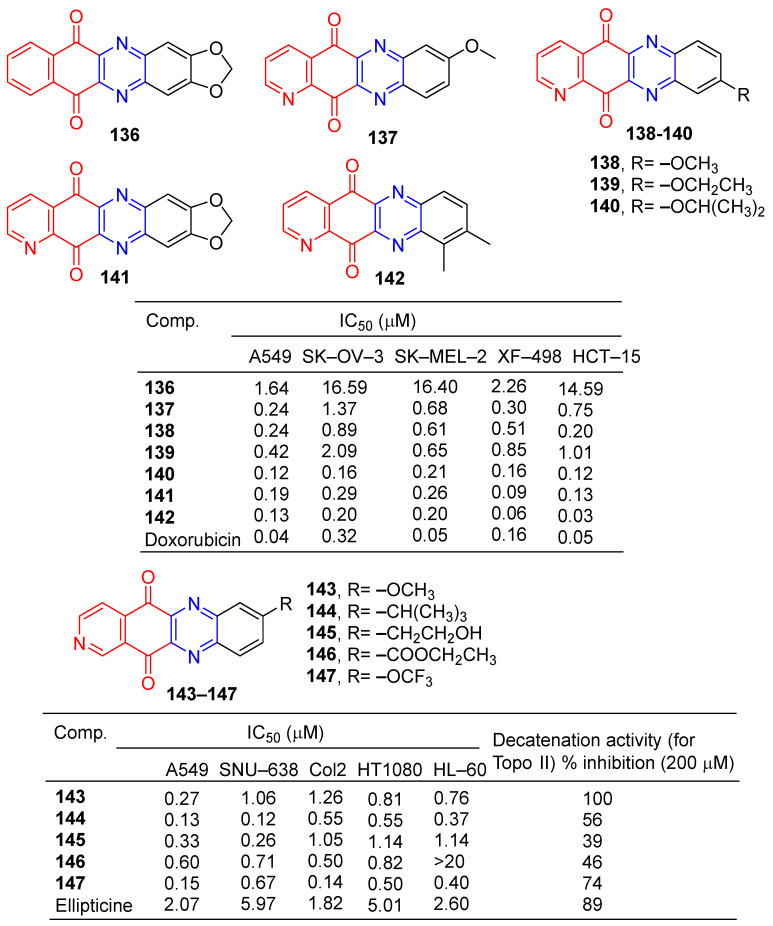
Anthraquinone–pyrazine derivatives **136**–**147**.

Kim et al. found that tetracyclic heteroquinone analogs containing pyrazine structures were highly cytotoxic to human tumor cell lines. Compound **136** (Figure 13) showed strong inhibitory effects on A549 and XF-498 cell lines with IC_50_ values of 1.64 and 2.26 μM. Compounds **137**–**142** showed significant inhibitory effects in A549, SK-OV-3, SK-MEL-2, XF-498, and HCT-15 cell lines, with IC_50_ values ranging from 0.06–1.01 μM. The IC_50_ value of compound **142** against the XF-498 cell line was 0.06 μM, 2.6 times that of doxorubicin (IC_50_ = 0.16 μM) [139].

Kim et al. designed and synthesized a series of pyrido [3,4-b] phenazinedione derivatives and evaluated their cytotoxic activity and topoisomerase II inhibitory activity. The derivative **143**–**147** showed significant inhibitory effects on human tumor cell lines (A549, SNU-638, Col2, HT1080, and HL-60), with IC_50_ values ranging from 0.12 to 1.26 μM. Compound **144** had the strongest effect on the SNU-638 cell line with an IC_50_ of 0.12 μM. It is 49.75 times that of ellipticine (IC_50_ = 5.97 μM). Compounds **143**–**147** showed inhibitory activity (39–100%) against topoisomerase II at 200 μM. The most active compound was **143**, with an IC_50_ of 0.082 μM [140].

**Figure 14:**
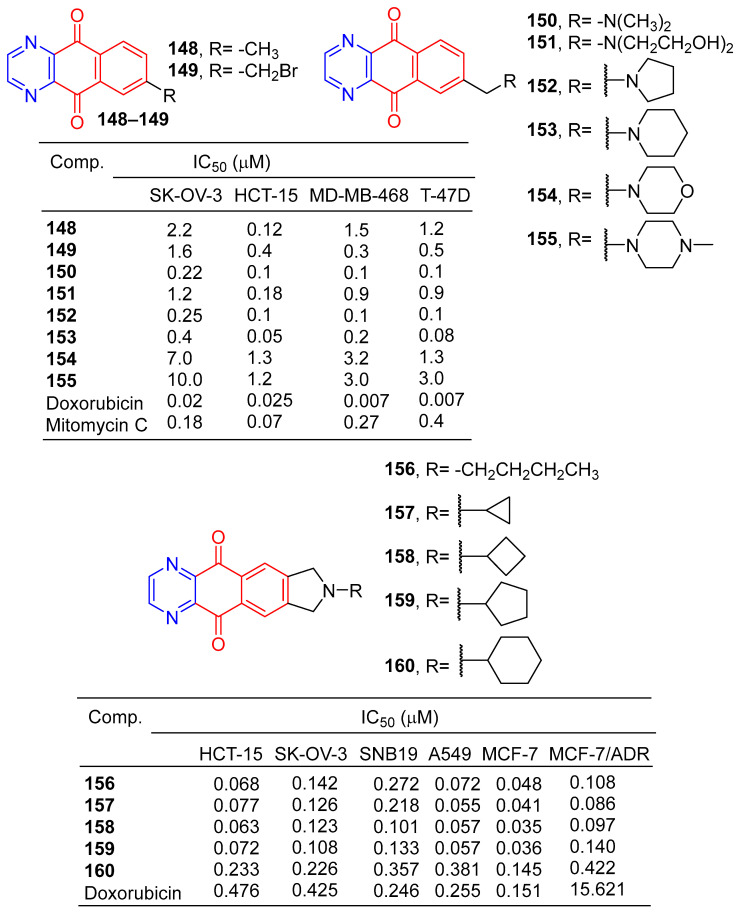
Anthraquinone–pyrazine derivatives **148**–**160**.

Lee et al. synthesized a series of benzo[g]quinoxalin-5,10-dione derivatives and evaluated in vitro cytotoxic activity against four human cancer cells (HCT-15, SK-OV-3, MD-MB-468, and T-47D). Compounds **148**–**155** (Figure 14) showed significant inhibitory activity against four cancer cells with IC_50_ values ranging from 0.005 to 10 μM. The cytotoxic activity of compound **153** against HCT-15 cells was similar to that of doxorubicin [141].

Kwak et al. synthesized a series of 2-alkyl-2, 3-dihydro-1h-2,6,9-triazacyclopenta[b]anthracene-5,10-diones. The cytotoxic activity of six human cancer cells (HCT-15, SK-OV-3, A549, SNB19, MCF-7, and MCF-7/ADR) was evaluated in vitro. Compounds **156**–**160** showed significant inhibitory effects on all human cancer cell lines, with IC_50_ values ranging from 0.035 to 0.381 μM [142].

**Figure 15:**
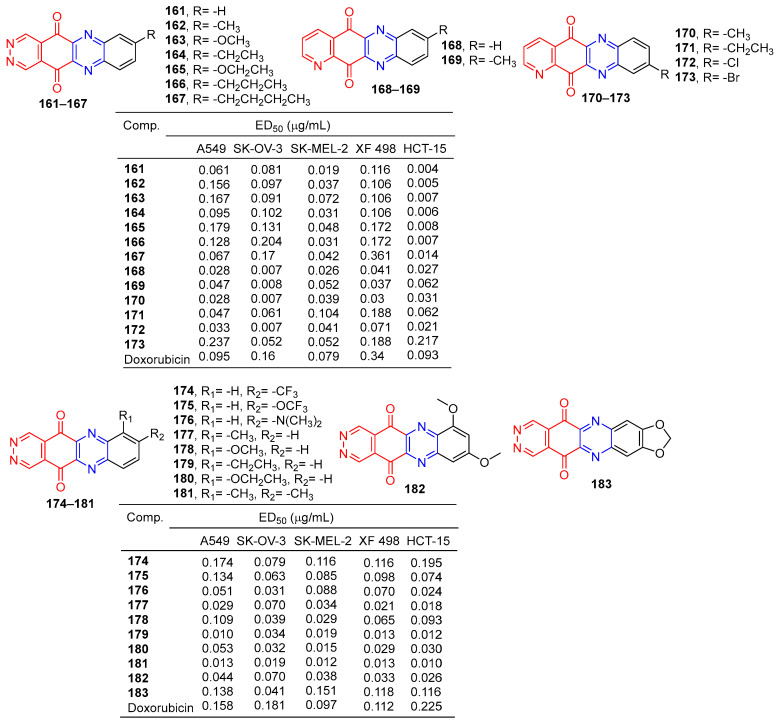
Anthraquinone–pyrazine derivatives **161**–**183**.

Lee et al. synthesized pyridazo [2,3-b] phenazine-6,11-dione derivatives and evaluated their cytotoxic activity by the SRB (Sulforhodamine B) assay. Derivatives **161**–**173** (Figure 15) showed excellent cytotoxicity to human tumor cell lines (A549, SK-OV-3, SK-MEL-2, XF-498, and HCT-15) with IC_50_ values ranging from 0.004–0.361 μg/mL. The killing effect of **161** on HCT-15 (ED_50_ = 0.004 μg/mL) was 23 times that of adriamycin (ED_50_ = 0.093 μg/mL) [143].

Lee et al. designed and synthesized pyridazino [4,5-b]phenazine-5,12-diones. The cytotoxic activity of these compounds against human cancer cell lines was evaluated by a SRB (thiodan B) assay. The cytotoxicity of compound **7a**–**7j** to cancer cells (A549, SK-OV-3, SK-MEL-2, XF498, and HCT-15) was higher (IC_50_ = 0.010–0.0.195 μM) than that of adriamycin (IC_50_ = 0.097–0.225 μM). The most active compounds **179** and **181** are about 10 times more cytotoxic than doxorubicin to all human cancer cell lines [144].

**Figure 16:**
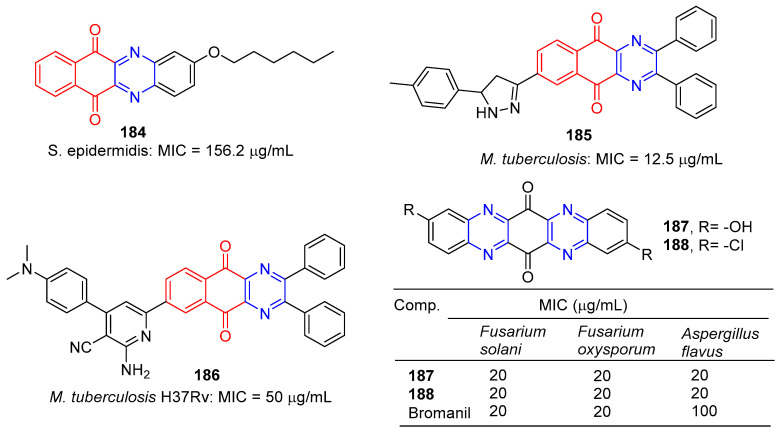
Anthraquinone–pyrazine derivatives **184**–**188**.

Tuyun et al. designed and synthesized a series of benzo[b]phenazine-6,11-dione derivatives and tested their antibacterial and antifungal activities in vitro. Among them, compound **184** (Figure 16) showed the strongest inhibition effect on *S. epidermidis*, and the MIC value was 156.2 μg/mL [145].

Kumar et al. designed and synthesized a series of benzoquinolin-5,10-dione compounds to test for in vitro antituberculosis activity against *M. tuberculosis* H37Rv. Compound **185** is the most active against *M. tuberculosis*, with a MIC of 12.5 μg/mL [146].

Kumar et al. designed and synthesized 2-amino-6-(5,10-dioxo-2,3-diphenyl-5,10-dihydrobenzo[g]quinoxalin-7-yl)-4-(substituted)phenylpyridine-3-carbonitrile. The antibacterial activity of newly synthesized compounds was screened by the L.J. Slope (conventional) method. Compound **186** has the strongest inhibitory effect against M. tuberculosis H37Rv with a MIC of 50 μg/mL [147].

S. Hammam and others designed and synthesized a series of diarylaminodiaminobenzoquinone, and studied the antifungal and antibacterial activities. Among them, compounds **187** and **188** had significant inhibitory effects on *Fusarium solani*, *Fusarium oxysporum*, and *Aspergillus flavus*, with MIC values of 20 μg/mL [148].

**Figure 17:**
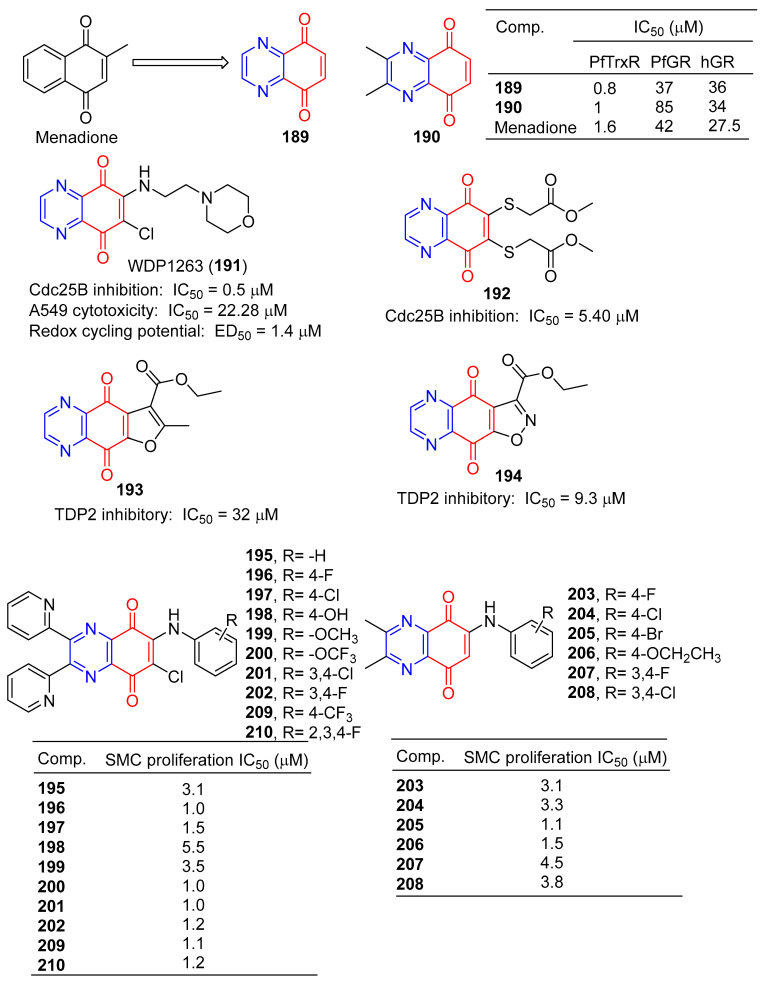
Anthraquinone–pyrazine derivatives **189**–**210**.

Morin et al. synthesized a series of various azotized analogs of 1, 4-naphthoquinone, The inhibitory activity of *P. falciparum* and human glutathione reductases and *P. falciparum* thioredoxin reductase was tested. Compounds 5,8-quinoxalinedione (**189**) and **190** (Figure 17) were the most specific TrxR inhibitors, with a stronger inhibitory effect than menadione [149].

The inhibitory activity of 5,8-quinolinedione (**189**) on the binding of BMAL1/CLOCK to Ebox DNA was concentration dependent, with an IC_50_ value of approximately 1 μM. 5,8-quinolinedione covalently reacts with protein(s) and may regulate dimer formation [150].

Keinan et al. designed and synthesized a series of Cdc25B quinone inhibitors. Among them, WDP1263 (**191**) containing pyrazine is the strongest Cdc25 inhibitor with an IC_50_ value of 0.5 μM, but in the presence of 0.8 mM DTT (EC_50_ = 1.4 μM). WDP1263 (**191**) showed inhibitory activity against the A549 cell line with an IC_50_ of 22.28 μM. WDP1263 (**191**) (E_1/2_ = 186 mV) prevents the redox cycle through its reducing state [151].

Besset et al. designed and synthesized a heteroquinone compound containing two methoxycarbonyl methyl sulfur groups in the benzoquinone ring and evaluated its Cdc25B phosphatase inhibitory activity. Compound **192** containing pyrazine showed a strong inhibitory effect on Cdc25B with an IC_50_ value of 5.40 μM. Furthermore, derivative **192** inhibited the pancreatic cell line (MiaPaCa-2) by 24% at 100 µM [152].

Yang et al. synthesized a series of furanoquinolinedione and isooxazolinequinolinedione derivatives and performed enzyme inhibition tests. Compounds **193** and **194** containing pyrazine have inhibitory activity of TDP2 with IC_50_ of 32 and 9.3 μM [153].

Ryu et al. reported that derivatives of 6-arylamino-quinoxalin-5,8-diones had inhibitory effects on the proliferation of rat aortic smooth muscle cells (RAoSMC). Compounds **195**–**208** significantly inhibited SMC proliferation, with IC_50_ values ranging from 1.0–5.5 μM. Compounds **196**, **200**, and **201** were the most active with IC_50_ values of 1.0 μM [154].

Chung et al. also reported that the 6-arylamino-quinoxalin-5,8-diones derivatives **195**–**202** and **209**–**210** had inhibitory effects on the proliferation of rat aortic smooth muscle cells (RAoSMC). The activity of compounds **195**–**202** was consistent with the literature. Additionally, compounds **209** and **210** significantly inhibited SMC proliferation with IC_50_ values of 1.1 and 1.2 μM. Furthermore, the inhibitory effect of compound **197** on SMC proliferation is mediated by the regulation of the kinase 1/2 signaling pathway regulated by extracellular signals [155].

**Figure 18:**
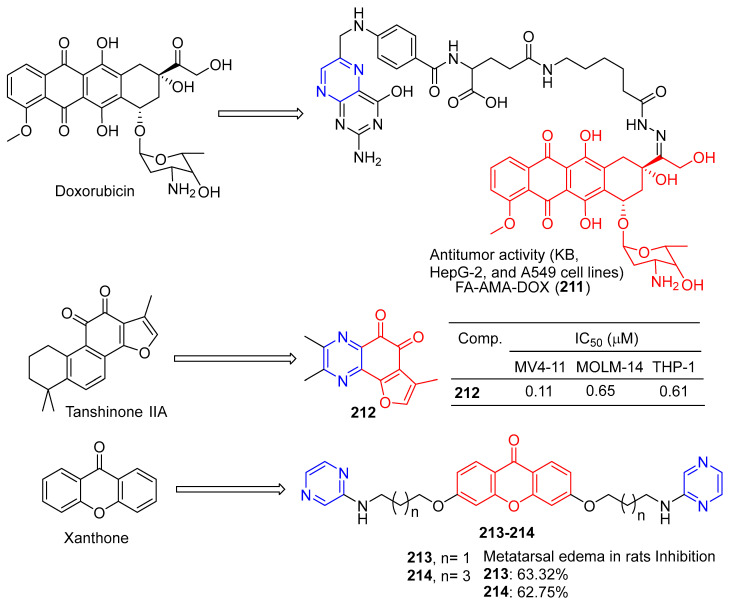
Structure of FA-AMA-DOX (**211**), *O*-naphthoquinone **212**, and xanthone derivatives **213**–**214**.

Ye et al. synthesized folate–aminocaproate–doxorubicin (FA-AMA-DOX) and performed cytotoxicity and uptake tests on KB, HepG-2, and A549 cell lines. FA-AMA-DOX (**211**) (Figure 18) is more cytotoxic to KB and HepG-2 cells than DOX or AMA-DOX at the same concentration, and FA can reduce cytotoxicity in a dose-dependent manner. On the contrary, FA-AMA-DOX and AMA-DOX showed lower cytotoxicity to A549 cells than DOX at the same concentration, and FA could not reduce cytotoxicity. FA-AMA-DOX (**211**) increased DOX accumulation in KB cells compared to FA-AMA [156].

Huang et al. oxidized the phenol to *O*-naphthoquinone and tested its biological activity. Compound **4h** effectively inhibited the proliferation of different AML (acute myelocytic leukemia) cell lines in vitro, with IC_50_ values ranging from 0.11 to 0.65 μM. In vivo antitumor studies have shown that compound **212** can cause tumor regression in MV4-11 xenograft tumor models at 40 mg/kg/d for 4 h, without obvious toxicity [157].

Sandilya et al. synthesized a series of xanthone derivatives containing 3,6-bis (3′-substituted propoxy) and 3,6-bis (5′-substituted pentyloxy). Anti-inflammation of Wistar albino rats was studied by carrageene-induced metatarsal edema in rats. Compounds **213** and **214** at 200 mg/kg body weight showed a slightly lower inhibitory effect than diclofenac sodium (10 mg/kg body weight dose, inhibition effect: 68.27%) in plantar edema after 6 h, with an inhibition effect of 63.32% and 62.75%, respectively [158].

### 2.7. Lignin–Pyrazine Hybridization

**Figure 19:**
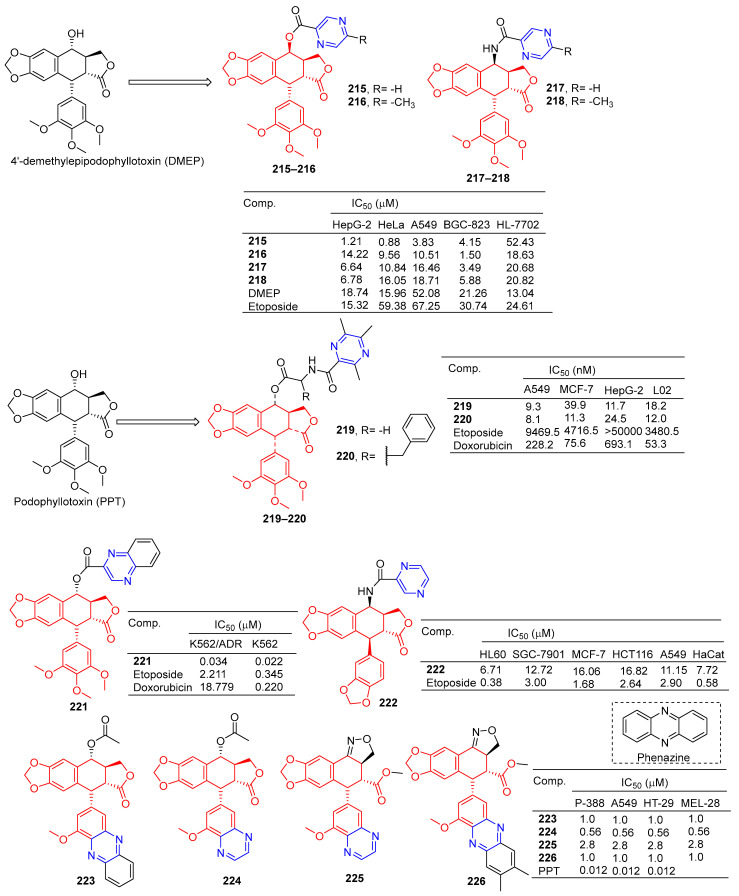
Lignin–pyrazine derivatives **215**–**226**.

According to Zhao et al., 4′-demethylepipodophyllotoxin (DMEP) was prepared by a series of new types of podophyllum topoisomerase II (Topo II) inhibitors. The antitumor activity of compound **215** (Figure 19) against the tumor cell lines HeLa, A549, HepG-2, and BGC-823 was significantly improved with IC_50_ values of 0.88, 3.83, 1.21, and 4.15 μM, respectively. More than 4′-demethylepipodophyllotoxin antitumor activity (the IC_50_ values of HeLa: 15.96 μM; HepG-2: 18.74 μM; A549: 52.08 μM; and BGC-823: 21.26 μM). The antitumor activity of compound **216** against BGC-823 was significantly improved with an IC_50_ value of 1.50 μM. The amide derivatives **217** and **218** showed strong inhibitory effects in HepG-2, HeLa, A549, and BGC-823 cell lines, with IC_50_ values ranging from 3.49 to 18.71 μM. Compound **217** had the strongest killing ability against the BGC-823 cell line with an IC_50_ value of 3.49 μM. Compound **215** inhibited the G2/M cycle of HeLa cells and induced apoptosis by strongly attenuating Topo II DNA unshackling relaxations [159]. 

Wu et al. synthesized a series of podophyllotoxin (PPT) derivatives and evaluated the cytotoxicity of A549, MCF-7, HepG-2, and L02 cells. The IC_50_ values of compound **219** containing pyrazine for A549 and HepG-2 cell lines were 9.3 and 11.7 μM. The IC_50_ values of compound **220** against the A549 and MCF-7 cell lines were 8.1 and 11.3 μM [160].

Zhang et al. synthesized a series of poxylotoxin aromatic heterocyclic esters and evaluated the anticancer effects of two human chronic myeloid leukemia cell lines (K562 and K562/ADR). The IC_50_ values of compound **221** containing pyrazine for the K562 and K562/ADR cell lines were 0.034 and 0.022 μM [161].

Li et al. designed and synthesized podophyllotoxin derivatives and evaluated their anticancer activity in vitro against several human cancers. The pyrazinyl derivative **222** inhibited the HL60, SGC-7901, and A549 cell lines with IC_50_ values of 6.71, 12.72, and 11.15 μM [162].

Castro et al. designed and synthesized podophyllotoxin e-ring-modified derivatives and evaluated their cytotoxicity. The IC_50_ value of compound **223**–**226** containing pyrazine against the P-388, A549, HT-29, and MEL-28 cell lines was 0.56–2.8 μM, and the antitumor activity of compound **223–226** was lower than that of podophyllotoxin (IC_50_ = 0.012 μM) [163].

**Figure 20:**
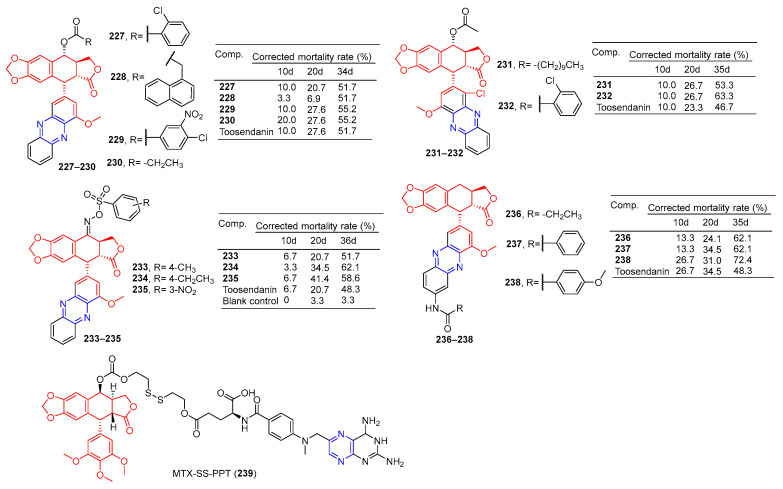
Lignin–pyrazine derivatives **227**–**239**.

Compounds **227**–**230** (Figure 20) had a corrected mortality rate of 51.7%, 51.7%, 55.2%, and 55.2% in vivo against *Mythimna separata* (*M. separata*) at 1 mg/mL, higher than or equivalent to the activity of Toosendanin (51.7%) [164].

Zhi et al. conducted an in vivo insecticidal activity test on the pre-third-instar larva of *M. separata* (Walker) at 1 mg/mL. Compounds **231** and **232** exhibited the best potent insecticidal activity with the final mortality rate of 53.3% and 63.3%, the activity was higher than Toosendanin (46.7%) [165].

Zhi et al. synthesized C-ring, D-ring, and E-ring modified phenazines oxme derivatives of podophyllotoxin and performed 1 mg/mL in vivo insecticide on the pre-third-instar larva of the oriental armyworm *M. separata* (Walker). Compounds **233**–**235** exhibited the best potent insecticidal activity with a final mortality rate of 51.7%, 62.1%, and 58.6%, the activity was higher than Toosendanin (48.3%) [166].

In vivo insecticidal activity against the pre-third instar larva of *M. separata* (Walker) was measured at 1 mg/mL. Derivatives **236**–**238** exhibited the most promising insecticidal activity with the final mortality rate of 62.1, 62.1%, and 72.4%, The activity was higher than toosendanin (48.3%). Depending on the symptoms of *M. separata* tested, the derivative **238** May shows anti-melting hormone effects [167].

Hou et al. connected methotrexate (MTX) with the hydrophobic drug podophyllotoxin (PPT) via a disulfide bond to obtain the amphiphilic drug–drug coupling prodrug (MTX-SS-PPT). The first two parent molecules of the drug self-assemble into stable nanoaggregates (NAs) in an aqueous solution, which realizes the self-delivery of the drug. Additionally, the presence of disulfide bonds in MTX-SS-PPT (**239**) can be controlled by using high concentrations of dithiothreitol (DTT). Intracellular mercaptan breaks disulfide bonds in MTX-SS-PPT (**239**), releasing drugs and killing tumor cells. Methotrexate-covered NAs can also target folate receptor-positive KB cells. Animal experiments have shown that methotrexate-covered NAs prodrug has good blood compatibility, and MTX-SS-PPT (**239**) NAs can reduce the size of xenograft tumors with few side effects [168].

### 2.8. Steroidal–Pyrazine Hybridization

**Figure 21:**
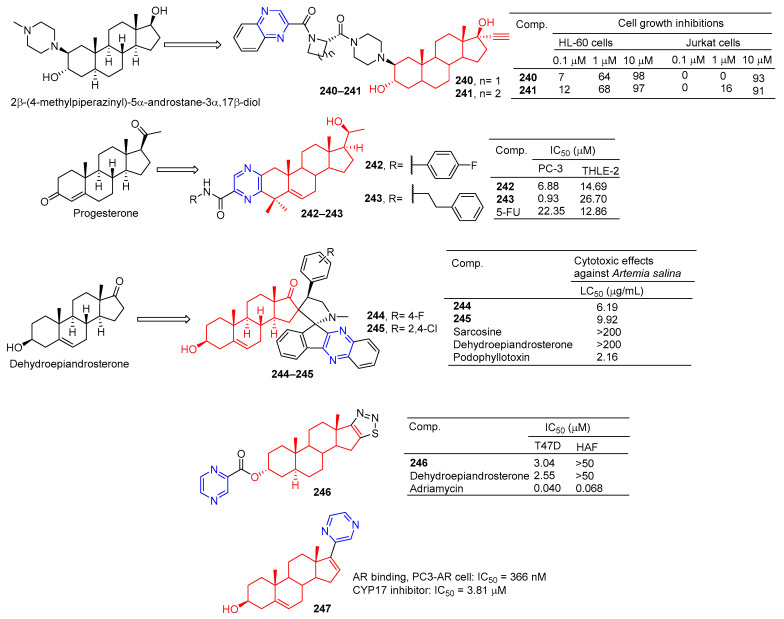
Steroidal–pyrazine derivatives **240**–**247**.

Amelie Talbot et al. designed and synthesized acetyne-based steroid derivatives **240** and **241** (Figure 21) and evaluated the antitumor activity of these two compounds. The inhibition rates of compounds **240** and **241** reached 98% and 97% at the concentration of 10 μM. The inhibition rates of Jurkat cells reached 93% and 91% at the same concentration [169].

Compounds **242** and **243** showed significant inhibitory effects on PC-3 cell lines, with IC_50_ values of 6.88 and 0.93 μM. THLE-2 cells of compound **243** showed low cytotoxicity (IC_50_ = 26.70 μM, SI = 28.71). Compound **243** induced apoptosis of PC-3 cells in a dose-dependent manner and led to cell cycle stagnation in the G2/M phase [170].

Tao et al. reported the synthesis and antitumor activity of DHEA derived from C-16 ropyrrolidine. Compounds **244** and **245** showed the best activity with LC_50_ values less than 6.19 and 9.92 µg/mL, exceeding dehydroepiandrosterone activity (LC_50_ > 200 µg/mL) by using the brine shrimp test [171].

The D-ring fused 1,2,3-thiadiazole dehydroepiandrosterone derivative **246** showed moderate inhibitory activity in T-47D cells with an IC_50_ value of 3.04 μM. Compound **246** was not as active as dehydroepiandrosterone (IC_50_ = 2.55 μM) [172].

Steroidal C-17 pyrazine (**247**) showed moderate inhibitory activity against PC3-AR cell lines with IC_50_ of 366 nM. Compound **247** is also a potent CYP17 inhibitor with an IC_50_ value of 3.81 μM for CYP17 [173].

**Figure 22:**
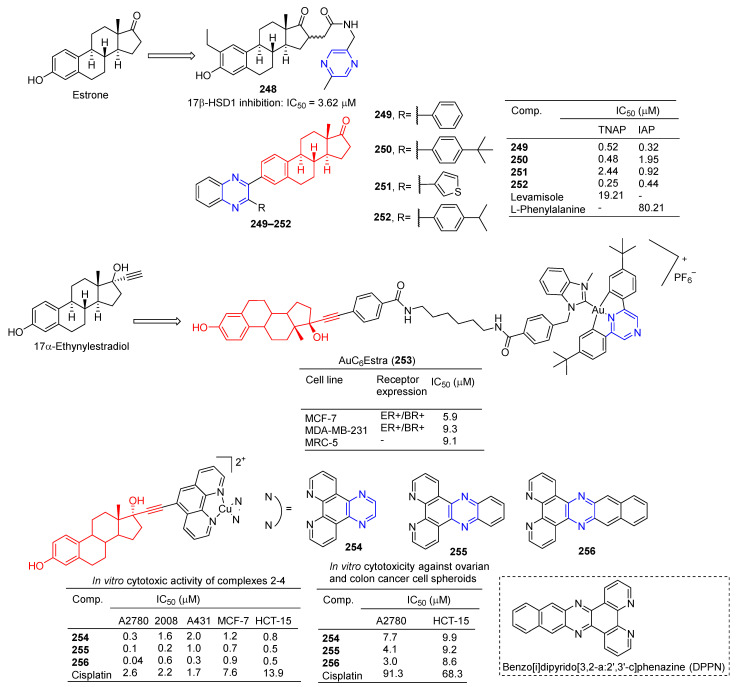
Steroidal–pyrazine derivatives **248**–**256**.

The 16-position aryl or heteraryl side chain of estrone is a potent inhibitor of 17β-HSD1. Among them, compound **248** (Figure 22) containing the pyrazine group showed an inhibitory effect on 17β-HSD1 with an IC_50_ value of 3.62 μM [174].

Ivanov et al. prepared a series of estrone-derived quinolines. Acetylenated estrone and its derivatives have significant biological activity as alkaline phosphatase inhibitors. Compounds **249**, **250**, and **252** were more potent TNAP inhibitors with IC_50_ values of 0.52, 0.48, and 0.25 µM, exceeding the activity of Levamisole (IC_50_ = 19.21 µM). Compounds **249**, **251**, and **252** are potent IAP inhibitors with IC_50_ values of 0.32, 0.92, and 0.44 µM, which exceeds the activity of L-phenylalanine (IC_50_ = 80.21 µM) [175].

Benoît et al. synthesized bimetallic Au(III)/Au(I) complexes with 17α-ethylestradiol as the carrier. The toxicity of estradiol-conjugated AuC6Estra (**253**) to estrogen receptor-positive (ER+) cancer cells was greater than that of ER-cancer cells and non-cancer cells. AuC6Estra (**253**) tested MCF-7 (ER+), MDAMB-231 (ER−), and MRC-5 (healthy fibroblasts) cells. The anti-proliferation effect of AuC6Estra (**253**) on ER+ cells was slightly higher than that in ER- and non-cancer cells [176].

Ananthan et al. synthesized a series of estrogen-functionalized copper complexes and studied them as electrochemically active DNA binding and splitting agents. The cytotoxic activity of these compounds was evaluated against estrogen receptor-positive (ER+) and negative (ER−) human cancer cell lines, and compounds **254**–**256** showed inhibitory effects against A2780, 2008, A431, MCF-7, and HCT-15 cell lines with IC_50_ values ranging from 0.04–2.00 μM. Complex **256** has a high intercalation interaction with nuclear DNA in vitro and is a strong DNA-cutting agent. Finally, complex **256** is involved in cellular redox stress by stimulating ROS production [177].

**Figure 23:**
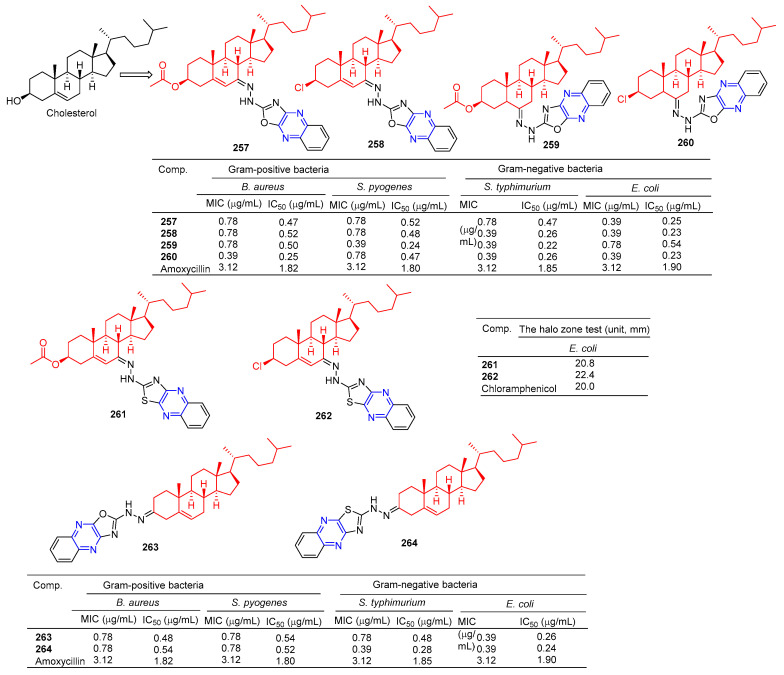
Steroidal–pyrazine derivatives **257**–**264**.

The steroid–pyrazine derivative **257**–**260** (Figure 23) showed significant antibacterial activity against two Gram-positive bacteria and two Gram-negative bacteria. The MIC value of compound **257** against the *E. coli* strain was 0.39 μM. The MIC value of compound **258** against the *S. typhimurium* and *E. coli* strains was 0.39 μM. The MIC value of compound **259** against the *S. pyogenes* strain was 0.39 μM. The MIC value of compound **260** against the *B. aureus*, *S. typhimurium*, and *E. coli* strains was 0.39 μM. The antibacterial activity of compound **257**–**260** was higher than that of the standard drug amoxicillin (MIC = 3.12 μM) [178].

Salman Ahmad Khan et al. found that the MIC values of compounds **261** and **262** against *E. coli* strains were 64 and 32 mg/mL, higher than the activity of cholesterol (MIC = 512 mg/mL). It was comparable to positive control chloramphenicol (MIC = 32 mg/mL) [179].

Khan et al. found that compounds **263** and **264** had significant antibacterial activity against two Gram-positive and two Gram-negative bacteria. The MIC values of compound **263** for *S. aureus*, *S. pyogenes*, *S. typhimurium*, and *E. coli* strains were 0.78, 0.78, 0.78, and 0.39 mg/mL. The MIC values of compound **264** were 0.78, 0.78, 0.39, and 0.39 mg/mL [180].

**Figure 24:**
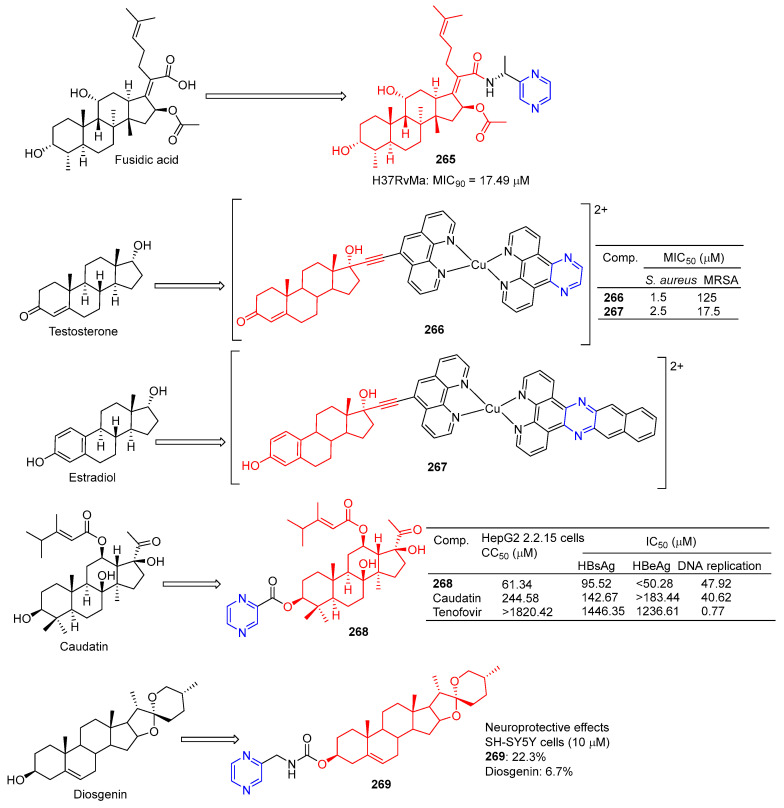
Steroidal–pyrazine derivatives **265**–**269**.

Compound **265** (Figure 24) showed moderate inhibitory activity against H37RvMa in MB7H9/ADC medium with a MIC_90_ value of 17.49 μM and low toxicity to CHO cells (IC_50_ > 50 μM) [181].

Stephen Barrett et al. used planephroline-modified aromatic ligands and copper (II) complexes of steroids (ethinynoestradiol and ethyl ketone) and screened these compounds for antimicrobial resistance against Staphylococcus aureus and methicillin-resistant *Staphylococcus aureus* (MRSA). Testosterone derivative **266** showed the strongest inhibitory effect on *S. aureus*, with a MIC_50_ of 1.5 μM. Estradiol derivative **267** showed the strongest inhibitory effect on MRSA, with a MIC_50_ of 17.5 μM [182].

Wang et al. synthesized the gaudatin–pyrazine derivative **268** and evaluated its anti-hepatitis B virus (HBV) activity in HepG-2 cells. Compound **268** not only inhibited the secretion of HBsAg (IC_50_ = 95.52 μM) and HBeAg (IC_50_ < 50.28 μM), but also inhibited the replication of HBV DNA (IC_50_ = 47.92 μM). Compound **268** showed low toxicity to HepG-2 cells, with a value of CC_50_ of 61.34 μM [183].

Compound **269** showed a neuroprotective effect on H_2_O_2_-induced SH-SY5Y cells, with a cell protective activity of 22.3% at 10 µM, more than diosgenin activity (6.7%) [184].

### 2.9. Terpene–Pyrazine Hybridization

**Figure 25:**
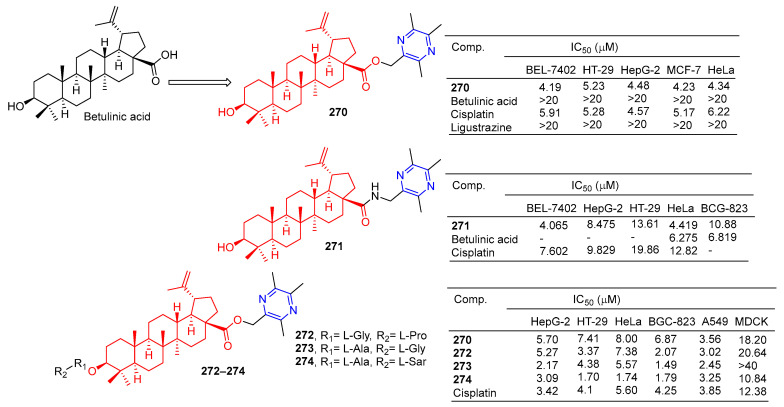
Betulinic acid–pyrazine derivatives **270**–**274**.

Betulinic acid-linked ligustrazine derivative **270** (Figure 25) showed good antitumor activity, with IC_50_ values of 4.19, 5.23, 4.48, 4.23, and 4.34 μM against BEL-7402, HT-29, HepG-2, MCF-7, and HeLa cells. The cytotoxicity selective assay showed that **270** had low cytotoxicity to MDCK cells (IC_50_ > 20 μM). Fluorescence staining and flow cytometry analysis showed that **270** could induce HepG-2 cell apoptosis. Further studies showed that **270**-induced apoptosis was mediated by depolarizing mitochondrial membrane potential and increasing intracellular free Ca^2+^ concentration [185].

The IC_50_ values of compound **271** against the BEL-7402, HepG-2, and HeLa cell lines were 4.065, 8.475, and 4.419 μM [186].

Xu et al. designed and synthesized a series of betulinic acid-linked ligustrazine derivatives, and screened their selective cytotoxic activity against five cancer cell lines (HepG-2, HT-29, HeLa, BCG-823, and A549) and nonmalignant cell lines MDCK using a standard MTT assay. Compounds **272** and **273** showed the strongest inhibitory effect on the BGC-823 cell line, with IC_50_ values of 0.84 and 1.49 μM. Compound **274** showed the highest cytotoxic activity against tumor cell lines (mean IC_50_ = 2.31 μM), and the strongest cytotoxic activity against HT-29 and HeLa with IC_50_ values of 1.70 and 1.74 μM. Further mechanism studies showed that **274**-induced apoptosis was associated with the loss of mitochondrial membrane potential and increased intracellular free Ca^2+^ concentration [187].

**Figure 26:**
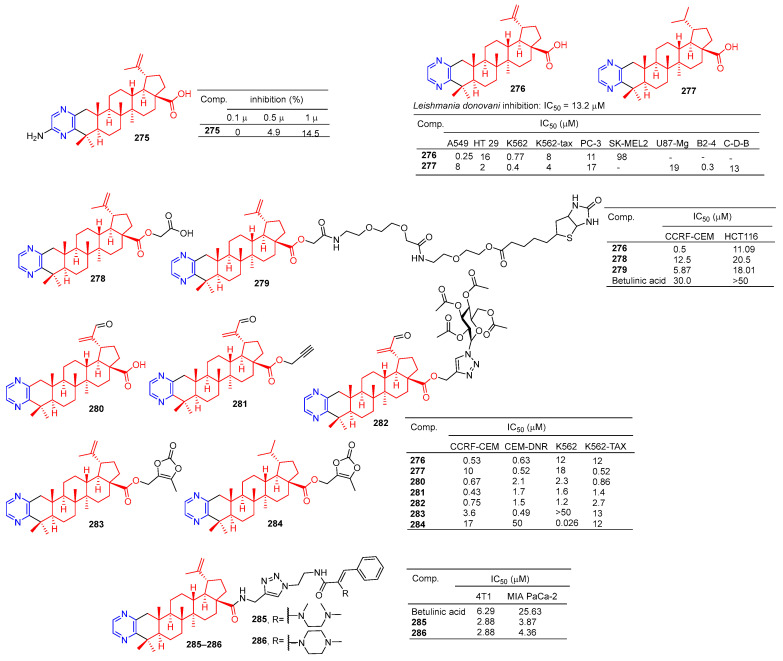
Betulinic acid–pyrazine derivatives **275**–**286**.

The betulinic acid derivative **275** (Figure 26) inhibited the osteoclast differentiation of RAW.264.7 cells induced by RANKL, and the inhibitory rate reached 100% at 5 μM. The activity of betulinic acid was higher than that of betulinic acid (5 μM: 0%). Compound **275** still had a certain inhibitory effect at a concentration of 1.0 μM, and the inhibitory rate was 14.5% [188].

Compound **276** showed an obvious inhibitory effect in the A549 cell line with an IC_50_ value of 0.25 μM. Compound **277** showed the strongest inhibitory effect on HT-29, K562, K562-TAX, and B2-4 cell lines with IC_50_ values of 2, 0.4, 4, and 0.3 μM [189].

Haavikko et al. determined the leishmania activity of compound **276** using the alamarBlue aseptic flagellate activity assay against leishmania donovani. Compound **276** showed an obvious inhibitory effect on leishmania donovani with an IC_50_ value of 13.2 μM [190].

The betulinic acid derivatives **276**, **278**, and **279** showed significant inhibitory effects on two cancer cells (CCRF-CEM and HCT116). The IC_50_ values of compound **276** against CCRF-CEM and HCT116 cell lines were 0.5 and 11.06 μM. The IC_50_ values of compound **278** against the CCRF-CEM and HCT116 cell lines were 12.25 and 20.5 μM. The IC_50_ values of compound **279** against CCRF-CEM and HCT116 cell lines were 5.87 and 18.01 μM [191].

Hodon et al. synthesized a series of betulinic acid–pyrazine compounds and tested the cytotoxicity of these compounds in multiple cancer cell lines. Compounds (**276–277** and **280**–**284**) were preferentially and highly cytotoxic to leukemia cell lines (CCRF-CEM, K562, CEM-DNR, and K562-TAX) (IC_50_ between 0.43 and 18 μM). Compound **283** showed a significant inhibitory effect in CIM-DNR cells with IC_50_ of 0.49 μM. The IC_50_ activity of compound **284** against K562 cells was 0.026 μM. In addition, compounds **276**, **280**, and **282** inhibited the growth and degradation of HCT116 and HeLa cells in sphere cultures [192].

Compounds **285** and **286** have an obvious inhibition effect on 4T1 and MIA-PaCa-2 cell lines. The IC_50_ value of compounds **285** and **286** against 4T1 cell lines was 2.88 μM, which exceeded the activity of betulinic acid (IC_50_ = 6.29 μM). The IC_50_ values of MIA-PaCa-2 cell lines were 3.87 and 4.36 μM, which exceeded the activity of betulinic acid (IC_50_ = 25.63 μM) [193].

**Figure 27:**
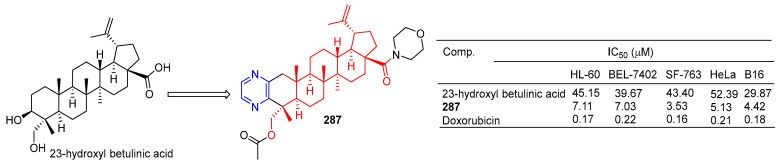
A 23-hydroxyl betulinic acid–pyrazine derivative **287**.

Pyrazine-thickened 23-hydroxyl betulinic acid, further modified by replacing C-28 carboxyl with ester and amide bonds, increased its antitumor activity. Compound **109** (Figure 27) showed the strongest activity against the cell lines SF-763, B16, and HeLa, with IC_50_ values of 3.53, 4.42, and 5.13 µM, respectively. In a preliminary mechanism study, **109** induced G1 phase cell arrest and significantly induced apoptosis of B16 cells in a dose-dependent manner. Furthermore, the in vivo antitumor activity of **109** was demonstrated in mice with H22 liver cancer and B16 melanoma (tumor inhibition rates were 55.6% and 62.7%, respectively) [194].

**Figure 28:**
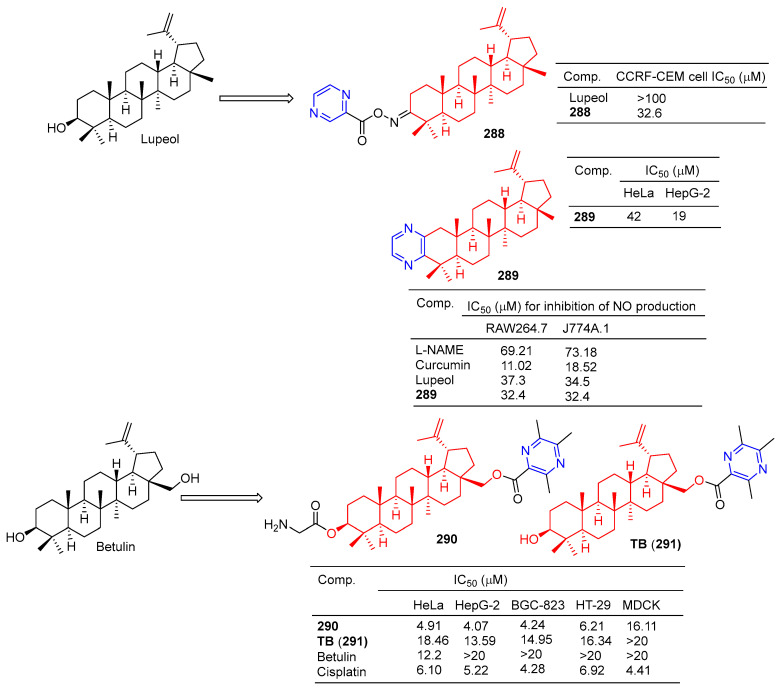
Lupeol–pyrazine derivatives **288**–**289** and betulin–pyrazine derivatives **290**–**291**.

Compound **288** (Figure 28) showed moderate antitumor activity, with GI_50_ values of 32.6 µg/mL for IMR 32 (neuroblastoma) cell lines [195].

Betulinic acid–pyrazine derivative **289** showed significant anti-proliferation activity against HeLa and HepG-2 cell lines with IC_50_ values of 42 and 19 μM [196].

Bhandari et al. synthesized the pyrazine derivative **289** from lupinol and evaluated its anti-inflammatory activity by inhibiting NO production in LPS-induced RAW264.7 and J774A.1 cells. The IC_50_ of compound **289** inhibited NO production in RAW264.7 and J774A.1 cells was 32.4 μM [197].

The cytotoxicity of compound **290** in cancer cells (mean IC_50_ = 4.86 μM) was three times higher than that of normal cells (mean IC_50_ = 16.11 μM). The IC_50_ values of compound **290** against the HeLa, HepG-2, BGC-823, and HT-29 cell lines were 4.91, 4.07, 4.24, and 6.21 μM. Additionally, **290** was more cytotoxic to tumor cells than the positive drug cisplatin. Furthermore, **290** was more cytotoxic to tumor cells than its lead compound TB (**291**) and positive control cisplatin. Subsequently, fluorescence staining, apoptosis detection, and cell cycle analysis showed that **290** induced early apoptosis of HepG-2 cells and blocked the G1 phase cell cycle [198].

**Figure 29:**
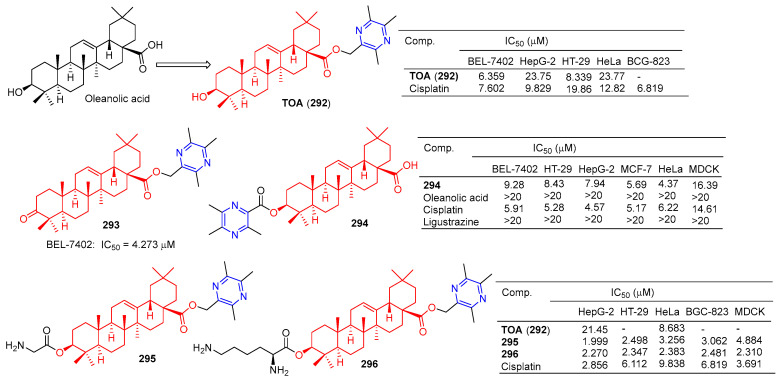
Oleanolic acid–pyrazine derivatives **292**–**296**.

The antitumor component ligustrazine was combined with oleanolic acid to form TOA (**292**) (Figure 29). TOA showed a good anticancer effect in vitro [79], with IC_50_ values of 21.45 and 8.683 μM for HepG-2 and HeLa cell lines [199]. The IC_50_ values of the BEL-7402, HepG-2, HT-29, and HeLa cell lines were 6.359, 23.75, 8.339, and 23.77 μM [186].

TOA (**292**) blocks the expression of the nuclear transcription factors NF-κB/p65 and COX-2 in S180 mice [200]. Furthermore, acute toxicity tests confirmed that the LD_50_ of TOA (**292**) exceeded 6.0 g/kg by intragastric administration in mice. However, the poor hydrophilicity of TOA (**292**) limits its oral bioavailability [200].

Compound **293** showed good antitumor activity, with an IC_50_ value of 4.273 μM against the BEL-7402 cell line [186].

Ursolic acid-linked ligustrazine derivative **294** showed good antitumor activity, with IC_50_ values of 9.28, 8.43, 7.94, 5.69, and 4.37 μM for BEL-7402, HT-29, HepG-2, MCF-7, and HeLa cells. The cytotoxicity selective assay showed that **294** had low cytotoxicity to MDCK cells (IC_50_ = 16.39 μM) [185].

Chu et al. linked amino acids to TOA (**292**) via ester bonds and evaluated their cytotoxicity in four cancer cell lines (HepG-2, HT-29, HeLa, and BGC-823) using a standard MTT assay. Compounds **295** and **296** not only showed good cytotoxicity (IC_50_ < 3.5 μM), but also showed better hydrophilicity than TOA (**292**). Compound **295** showed the strongest inhibitory effect on the HepG-2 cell line with an IC_50_ value of 1.999 μM. Compound **296** showed the strongest inhibitory effect on HT-29, HeLa, and BGC-823 cell lines with IC_50_ values of 2.347, 2.383, and 2.481 μM. Furthermore, the nephrotoxicity of 6a (IC_50_ = 4.884 μM) to MDCK cells was lower than that of **296** (IC_50_ = 2.310 μM) and cisplatin (IC_50_ = 3.691 μM). Combination **296** can induce HepG-2 apoptosis through nuclear division and has low nephrotoxicity [199].

**Figure 30:**
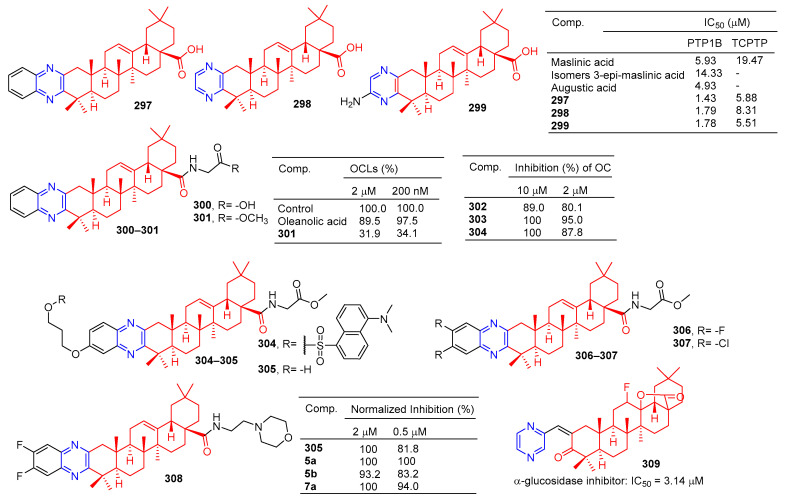
Oleanolic acid–pyrazine derivatives **297**–**309**.

A series of hawthorn acid derivatives were synthesized by introducing various thickened heterocycles at C-2 and C-3. Their inhibitory effects on PTP1B, TCPTP, and related PTPS were evaluated. Compounds **297**–**299** (Figure 30) showed significant increases in inhibitory power and selectivity, and the three most potent PTP1B inhibitors, **297** (IC_50_ = 1.43 μM), **298** (IC_50_ = 1.79 μM), and **299** (IC_50_ = 1.78 μM), were shown to be about two times stronger than hawkic acid. Furthermore, **297**–**299** are 4.1, 4.6, and 3.1 times more selective for PTP1B than for TCPTP [201].

Oleanolic acid has been found to have an anti-bone resorption effect. The oleanolic acid derivative **300** inhibited the formation of osteoclast-like multinucleated cells (OCL) and showed quite strong activity even at 200 nM. The formation of oleanolic acid was only 34.1% compared to the control group (100.0%) [202].

Compound **301** is a novel molecule with a strong anti-osteoporosis effect in vivo. To study the molecular mechanism of **301**, a novel fluorescent-labeled chemical probe with biological activity was designed and synthesized. Compared to **302**, fluorescence compounds **303** and **304** showed a stronger inhibitory activity against RANKL-induced osteoclast differentiation in RAW264.7 cells at 2 μM, with an inhibition rate of 95.0% and 87.8%. Compounds **303** and **304** did not show cytotoxicity for the RAW264.7 cell line at a concentration of 10 μM [118].

The inhibitory activity of pyrazine-fused oleanolic acid derivatives on osteoclast formation induced by the nuclear factor-κB ligand receptor activator (RANKL) was evaluated using a cell-based tartrate-resistant acid phosphatase (TRAP) assay. The most potent compound **305** had an IC_50_ of 62.4 nM, and cytotoxicity in marine-derived bone monocytes/macrophages (BMDMs) indicated that inhibition of **305** in osteoclast differentiation was not due to its cytotoxicity. More importantly, **305** mitigated bone loss in bilateral ovariectomy mice, and preliminary mechanism studies showed that **305** affected the early stages of osteoclast genesis [203]. Furthermore, compounds **305**–**308** showed considerable inhibitory activity, inhibiting osteoclast formation by more than 80% at lower concentrations (0.5 μM) compared to the control group.

The results of α-glucosidase inhibitory activity in vitro showed that compound **309** showed certain inhibitory activity with IC_50_ values of 3.14 μM, respectively. However, the activity was inferior to oleanolic acid (IC_50_ = 2.41 μM) [204].

**Figure 31:**
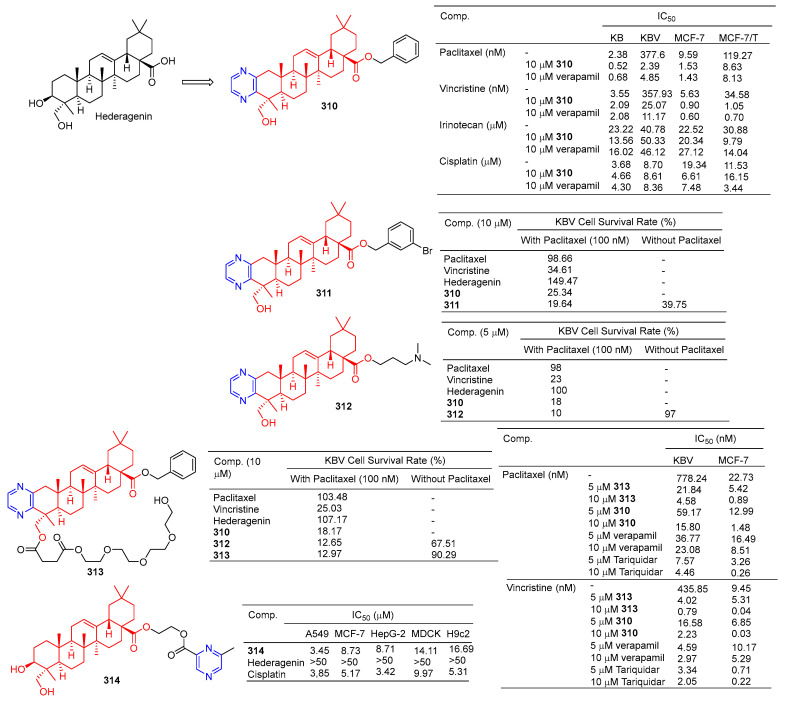
Hederagenin–pyrazine derivatives **310**–**314**.

Derivative **310** (Figure 31) is a hederagenin derivative that binds to paclitaxel at 10 µM with an IC_50_ value of 2.4 nM against drug-resistant KBV cells. Derivative **310** can activate P-gp ATPase, resulting in the inability of drug-resistant cells to remove the drug from the body. Therefore, the derivative **310** can enhance the antitumor activity of paclitaxel in KBV cells, and the reversal effect of the drug is stronger than that of verapamil (IC_50_ = 4.9 nM). Furthermore, in vivo experiments showed that under combined use of paclitaxel (30 mg/kg) and derivative **310** (10 mg/kg), the body weight of nude xenograft mice decreased slightly, and tumor weight decreased to 41.88%. The results showed that derivative **310** reversed multidrug resistance by stimulating the ATPase activity of P-gp and then competing with chemotherapeutic drugs for binding to P-gp, but was less soluble due to the benzyl group at C-28 [205].

The cell assay showed that derivative **311** had the strongest antitumor reversion activity. When the derivative **311** (10 μM) was combined with paclitaxel (100 nM), the survival rate of the KBV cells reached 18.60%, surpassing that of compound **310** (19.64%) and hederagenin (149.47%) [206].

Compound **312** at 5 µM significantly improved the cytotoxicity of paclitaxel in resistant KBV cells and sensitized cells to paclitaxel, thus preventing cells from entering the G2/M phase and inducing apoptosis. Compound **312** may block the efflux of P-gp drugs by stimulating the activity of P-gp ATPase. In vivo experiments demonstrated that compound **312** increased the efficacy of paclitaxel in KBV cancer cell-derived xenograft tumors [207].

In order to improve the water solubility and tumor multidrug resistance reversal activity of **309**, Wang et al. designed and synthesized a new series of hederagenin derivatives. These derivatives significantly reversed the multidrug resistance phenotype of KBV cells to paclitaxel at a concentration of 10 μM. The water solubility of PEGylated derivatives **313** increased approximately 20 fold compared to **310**, while maintaining tumor multidrug resistance reversal activity. Therefore, pegylation is an effective method to improve water solubility while maintaining tumor multidrug resistance reversal activity [208]. Compound **313**, the most active compound in vitro, showed good chemical stability to esterases within 24 h and increased the sensitivity of KBV cells to paclitaxel and vincristine with IC_50_ values of 4.58 and 0.79 nM, respectively. Compound **313** also increased the sensitivity of MCF-7T cells to paclitaxel and vincristine with IC_50_ values of 0.89 and 0.04 nM, respectively. The combination of compound **313** and paclitaxel significantly increased the apoptosis rate of KBV cells. Compound **313** treatment increased the accumulation of rhodamine 123 and Flutax1 in KBV and MCF-7T cells at 5 and 10 μM concentrations, suggesting that compound **313** played a role in reversing tumor resistance by effectively inhibiting the efflux function of P-gp [208].

Fang et al. designed and synthesized a series of hederagenin–pyrazine derivatives and screened the in vitro cytotoxicity of five tumor cell lines. The antitumor activity of compound **314** against A549 (IC_50_ = 3.45 µM) was comparable to that of cisplatin (IC_50_ = 3.85 µM). Compound **314** induced early apoptosis of A549 cells in a concentration-dependent manner and induced cell arrest in the S phase [209].

**Figure 32:**
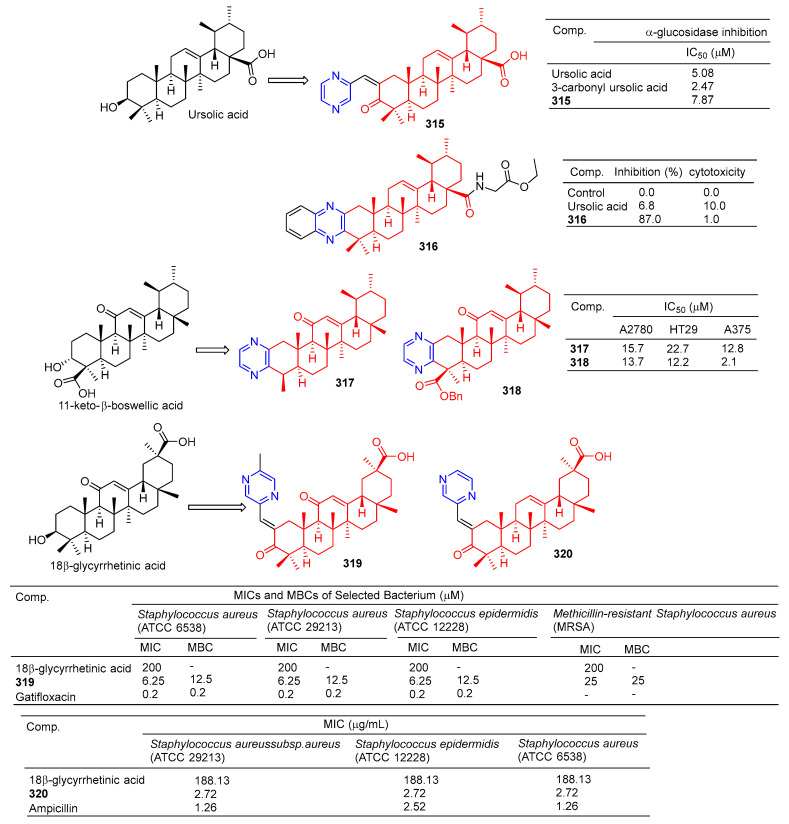
Other pentacyclic triterpenes–pyrazine derivatives **315**–**320**.

The results of α-glucosidase inhibitory activity in vitro showed that compound **315** (Figure 32) showed certain inhibitory activity with IC_50_ values of 7.84 μM, respectively. However, the activity was less than 3-carbonyl ursolic acid (IC_50_ = 2.47 μM) [210].

Tryptophan hydroxylase 1 (Tph-1) is the main enzyme in the biosynthesis of peripheral blood albumin, providing a new target for the design of anabolic agents for osteoporosis. Fu et al. synthesized a series of ursolic acid derivatives and bioevaluated them using RBL2H3 cells and ovariectomized rats. Among these compounds, compound **316** showed effective inhibitory activity against serotonin biosynthesis. Further studies showed that **316**, as an effective Tph-1 binder identified by SPR (estimated KD: 6.82 μM), inhibited the protein and mRNA expression of Tph-1 and reduced the serum and intestinal serotonin content, but had no effect on brain serotonin. In addition, in ovariectomized rats, oral administration of **316** increased serum levels of *N*-terminal propeptide (a marker of bone formation) of type 1 procollagen (P1NP) and improved bone microstructure without estrogenic side effects [211].

The pyrazine derivatives **317** and **318** of boswellic acid showed obvious antitumor effects. The IC_50_ values of compound **317** on A2780, HT-29, and A375 cells were 15.7, 22.7, and 12.8 μM. The IC_50_ values of compound **318** were 13.7, 12.2, and 2.1 μM [212].

Wu et al. synthesized 18β-glycyrtinic acid derivative **319** by introducing piperazine into C-2 after the hydroxyl group in C-3 was oxidized and evaluated its antibacterial activity. Compound **319** pairs of *Staphylococcus aureus* (ATCC 6538), *Staphylococcus aureus* (ATCC 29213), and the strain *Staphylococcus epidermidis* (ATCC 12228) showed obvious inhibition, and the MIC_50_ and MBC_50_ values were 6.25 and 12.5 μM, respectively [213].

Compound **320** showed a greater inhibitory effect on Gram-positive bacteria than glycyrrhetinic acid. Questions about *Staphylococcus aureussubsp aureus* (ATCC 29213), *Staphylococcus epidermidis* (ATCC 12228), and the MIC value of Staphylococcus aureus (ATCC 6538) was 2.72 μg/mL. The inhibitory activity was similar to that of ampicillin. In vivo, compound **320** was also shown to have anti-inflammatory effects, and 40.0 mg/mL gavage reduced approximately 59.69% of TPA-induced ear edema in mice. Immunohistochemical results showed that inhibition was related to inhibition of TPA-induced upregulation of the pro-inflammatory cytokines TNF-α and IL-1β. Furthermore, compound **320** significantly reduced the expression level of p65 in the NF-κB signaling pathway [214].

**Figure 33:**
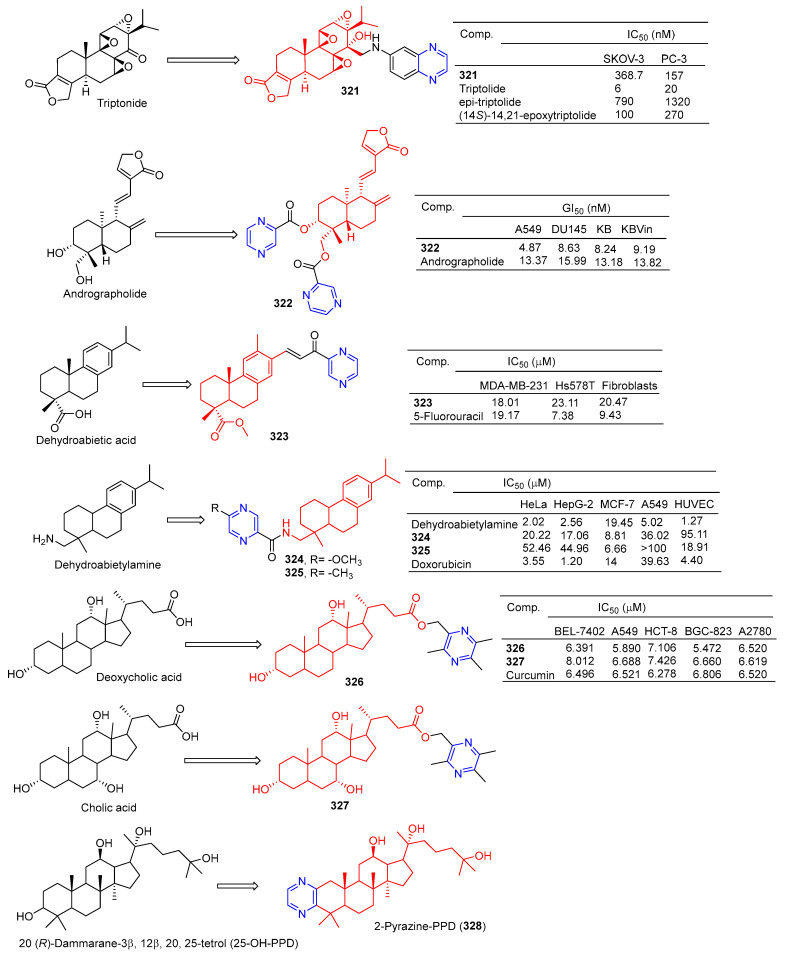
Other terpenes–pyrazine derivatives **321**–**328**.

Xu et al. synthesized a series of C14 heterocyclic substituted epi-triptolide derivatives. Among them, the pyrazine derivatives **321** (Figure 33) showed certain inhibitory effects on SKOV-3 and PC-3 cell lines, with IC_50_ values of 368.7 nM and 157 nM. The activity was higher than epitriptolide (SKOV-3, IC_50_ = 790 nM; PC-3, IC_50_ = 1320 nM) [215].

Wei et al. obtained derivative **322** by esterification and etherification of 14-dehydroxy-11,12-didehydroandrographolide. Compound **322** showed obvious inhibitory effects on A549, DU145, KB, and KBVin cell lines with IC_50_ values of 4.87, 8.63, 8.24, and 9.19 μM. The activity of compound **322** exceeded that of andrographolide (A549, IC_50_ = 13.37 μM; DU145, IC_50_ = 15.99 μM; KB, IC_50_ = 13.18 μM; KBVin, IC_50_ = 13.82 μM) [216].

Grigoropoulou et al. synthesized a series of dehydroabietic acid–chalketone heterozygotes. Compound **323** containing pyrazine showed certain inhibitory effects on MDA-MB-231 and Hs578T cell lines with IC_50_ values of 18.01 and 23.11 μM. However, compound **323** also showed some toxicity to fibroblasts with an IC_50_ value of 20.47 μM [217].

Zhao et al. synthesized a series of dehydroabietylamine derivatives containing pyrazine cycloheterocyclic rings. Anti-MCF-7 activity of compounds **324** and **325** increased (IC_50_: 8.81 and 6.66 μM) compared to dehydroabietylamine (IC_50_: 19.45 μM). However, the activity of **324** and **325** against other cells such as HeLa, HepG-2, A549, and HUVEC was lower than that of dehydroabietylamine [218].

Wang et al. designed and synthesized ligustrazine–deoxycholic acid/cholic acid derivatives as antitumor drugs. The IC_50_ values of compounds **326** and **327** against the BEL-7402, A549, HCT-8, BGC-823, and A2780 cell lines were 5.472–8.012 μg/mL [79].

Zhao et al. found that 2-Pyrazine-PPD (**328**) showed inhibitory activity in the gastric cancer BGC-823 cell line (IC_50_ = 11.52 μM). There is little toxicity to normal cells (human gastric epithelial cell line GES-1). Further studies showed that 2-Pyrazine-PPD (**328**) induced apoptosis of BGC-803 cancer cells through the mitochondrial pathway. Ros production was significantly increased in BGC-803 cancer cells treated with 2-Pyrazine-PPD (**328**). Therefore, 2-Pyrazine-PPD (**328**) exhibits anticancer activity through ROS-mediated apoptosis of gastric cancer cells and stress of the endoplasmic reticulum [219].

**Figure 34:**
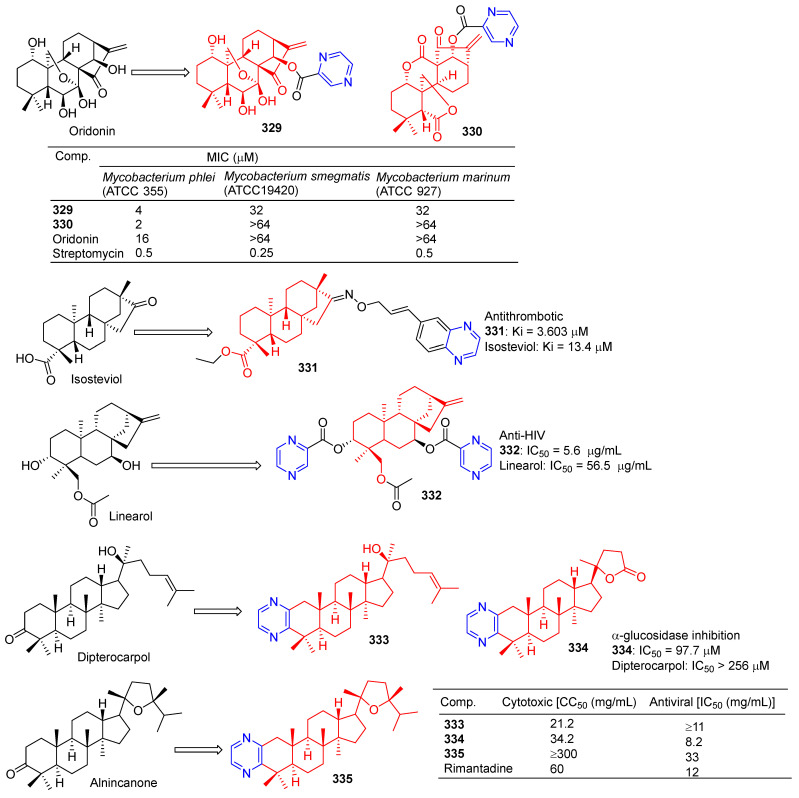
Other terpenes–pyrazine derivatives **329**–**335**.

Xu et al. introduced piperazine in C-14 to synthesize Rabbesin derivatives for **329** (Figure 34) and evaluated their antibacterial activity. Compound **329** showed significant inhibitory effects on *Mycobacterium phlei* (ATCC 355), *Mycobacterium smegmatis* (ATCC 19420), and *Mycobacterium marinum* (ATCC 927). The MIC_50_ values are 4, 32, and 32 μM [220].

Xu et al. synthesized **330**, an enmein derivative containing pyrazine, and studied its bacteriostatic effect. Compound **330** showed an obvious inhibitory effect on *Mycobacterium phlei* (ATCC 355) with a MIC_50_ of 2 μg/mL [221].

Chen et al. synthesized the C-16 carbonyl derivative **331** using isostevia as the raw material and evaluated its anticoagulant activity. In vitro activity of human FXa showed that **331** (Ki = 3.603 μM) showed relatively better inhibitory activity than isosteviol (Ki = 13.4 μM) [222].

Compound **332** showed obvious inhibitory activity against HIV replication in H9 lymphocytes with an IC_50_ value of 5.6 μg/mL more than linearol activity (IC_50_ = 56.5 μg/mL) [223].

Alla D. Zorina et al. tested the antiviral activity of pyrazine triterpenoids against the influenza virus A/Puerto Rico/8/34 (H1N1). The CC_50_ and IC_50_ values of compound **333** pairs of influenza virus A/Puerto Rico/8/34 (H1N1) were 21.2 and >11 μg mL^−1^. The CC_50_ and IC_50_ values of compound **334** were 34.2 and 8.2 μg mL^−1^. The IC_50_ values of compound **335** were 33 μg mL^−1^ [196].

The results of α-glucosidase inhibitory activity in vitro showed that compound **334** exhibited certain inhibitory activity, with IC_50_ values of 97.7 μM, respectively, which exceeded the activity of acarbose (IC_50_ = 397.6 μM) [224].

### 2.10. Alkaloid–Pyrazine Hybridization

**Figure 35:**
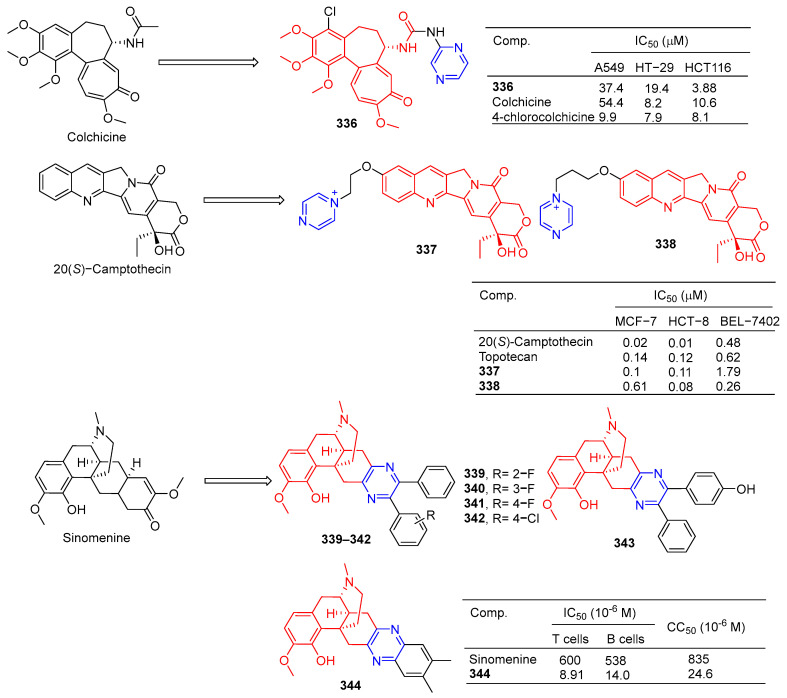
Alkaloid–pyrazine derivatives **336**–**344**.

Nishiyama et al. synthesized a 4-pyrazine substituted colchicine derivative **336** (Figure 35). Compound **336** showed moderate cytotoxicity against three human cancer cell lines (A549, HT-29, and HCT116), with IC_50_ values of 37.4, 19.4, and 33.0 μM [225].

In order to improve the antitumor activity of camptothecin, Li et al. designed and synthesized a series of 10 substituted camptothecin derivatives. Compound **337** showed the strongest inhibitory effect on the MCF-7 cell line with an IC_50_ value of 0.1 μM. Compound **338** showed the strongest inhibitory effect on HCT-8 and BEL-7402 cell lines with IC_50_ values of 0.08 and 0.26 μM [226].

Sinomenine derivatives showed stronger TNF-α inhibitory activity than sinomenine. Compounds **339**–**343** inhibited the production of TNF-α in mice peritoneal macrophages stimulated by LPS in vitro at a concentration of 10 μM, and the inhibitory rate was more than 95% [227].

Compound **344** inhibited TNF-α-induced NF-κB activation in a dose-dependent manner and showed a significant in vivo therapeutic effect on mice models of experimental autoimmune uveitis disease [228].

**Figure 36:**
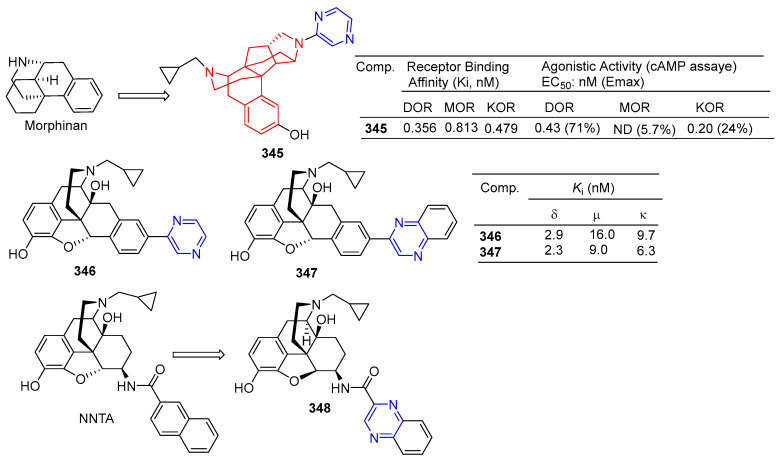
Alkaloid–pyrazine derivatives **345**–**348**.

Watanabe et al. reported on a morphinan derivative of the oxazatricyclodecane skeleton and tested its opioid receptor agonist activity. Pyrazine-containing compound **345** (Figure 36) showed a high affinity for all types of receptors (DOR, MOR, and KOR) [229].

Ananthan et al. synthesized pyridomorphinans with aromatic or heterocyclic substitutions at the 5′ position of the morphinan pyridine ring and evaluated the binding and functional activity of opioid receptors δ, μ, and κ. Pyrazine-containing compounds **346** and **347** show significant affinity for these three receptors, with Ki values ranging from 2.3 to 16 nM.

The spinal selective mu-kappa agonist NNTA selectively activates the mu-kappa isomer in HEK-293 cells and produces an unusually potent antipain response after administration in the mouse intrasheath (i.t.) [230]. The quinoline analog **348** of NNTA is a potent agonist (ED_50_ values of 50.76 and 757.2 pmol, respectively) with a 15-fold increase in spinal cord efficacy in both in vitro and in vitro perfusion pathways. In particular, **348** showed no significant tolerance to either mode of administration [231].

## 3. Conclusions

Natural products derived from microorganisms, plants, and animals are a rich source of effective drugs with significant structural diversity and biological properties, which offer the possibility for researchers to develop new molecules to treat disease [232]. Over the past few decades, drugs that directly or indirectly replace natural product derivatives and analogs have played an important role in the fight against disease [233]. Natural products have a wide range of biological activities, but their activity is not strong and often has the shortcomings of low bioavailability and poor solubility [234]. Therefore, to improve their physical properties and ADME, it is important to make the necessary structural modifications to them. Through a literature review, it is found that pyrazine stents have considerable biological relevance in anti-osteoporosis, antiviral, anti-diabetic, anti-inflammatory, anti-thrombotic, anti-parasitic, anti-malaria, antibacterial, anticancer, and other studies, leading to the emergence of many strong bioactive pyrazine compounds.

Although much progress has been made in the pharmacochemistry of natural product–pyrazine complexes, the following research opportunities remain to be further investigated: Since many studies have not reported detailed studies of structural modifications of lead compounds, a complete SAR study of natural product–pyrazine complexes is needed. Detailed SAR studies may reveal more active compounds. Furthermore, because pyrazines have a variety of biological activities and multitarget properties, most natural product–pyrazine derivatives do not have specific drug adaptability, hindering the development of these derivatives from laboratory to clinical applications; therefore, the in vivo activity of the natural product–pyrazine hybrid needs to be evaluated. Additionally, most natural products–pyrazine derivatives have no clear targets. To solve this problem, using reasonable drug design strategies such as computer-aided drug design, the target identification of a powerful natural product–pyrazine hybrid was studied, and the compounds were optimized. Finally, pharmacophore combinations and structure-based drug design strategies should be widely used in the following studies to develop more novel, more active, and more specific natural products–pyrazine derivatives, and the mechanism of action of these compounds should be studied in detail.

In conclusion, this review provides a favorable reference for the study of compounds containing pyrazine fragments. Pyrazine derivatives of natural products still have a wide range of research prospects and are worth further research and development. With the rapid development of combinatorial chemistry, rational drug design, and chemical proteomics, we believe that researchers will find more novel derivatives of pyrazines with good biological activity and wide application.

## Figures and Tables

**Table 1:** Examples of pyrazine-containing drugs and their pharmacological applications.

Drug	Structure	Biological Activity	Refs.
Acipimox	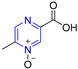	Hypolipidemic agent	[8]
Amiloride	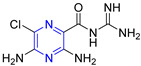	Potassium sparing diuretic	[9]
Benzamil	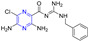	Potassium sparing diuretic	[10]
Bortezomib	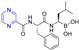	Proteasome inhibitor	[11,12,13]
Glipizide	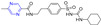	Anti-diabetic agent	[14]
Morinamide	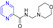	Anti-tubercular agent	[15]
Pyrazinamide	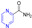	Anti-tubercular agent	[16]
Oltipraz	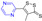	Schistosomicide and antitumor	[17]
Rimonabant (non-aryl derivative)	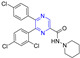	Cannabinoid receptor antagonist	[18]
Elpetrigine	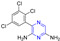	Antiepileptic	[19]
Verenicline	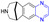	Used to treat smoking addiction	[20]
Zibotentan	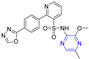	Anticancer agent	[21]
Amiloride	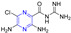	EnaC blocker	[22]
Paritaprevir	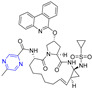	NS3-4A serine protease inhibitor hepatitis C treatment	[23]
Eszopiclone	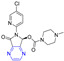	Insomnia	[24,25]
Zopiclone	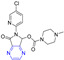	Treatment of sleep disorders	[26]
Cephalostatin 1	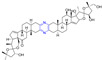	Anticancer	[27,28]
Favipiravir	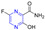	Antiviral (approved in Japan, influenza; FDA clearance, COVID-19)	[29,30,31]
(-)-Barrenazine A	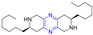	Anticancer	[32]
(-)-Barrenazine B	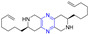	Anticancer	[32]
Alocasin A	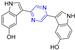	Anticancer	[33]
AKN-028	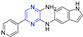	Acute myeloid leukemia	[34,35]
Botryllazine	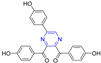	Anticancer	[36,37]
Phenazine-1-carboxylic acid	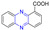	Antibacterial	[38]
2-Bromo-1-hydroxy phenazine	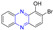	Antibacterial	[39]
Griseolutein A	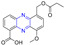	Antibacterial	[40]
HP-14	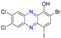	Biofilm-eradicating agent	[41]
Iodinin	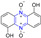	Antibacterial	[42]
Myxin	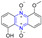	Antibacterial	[43]
Quinoxidine	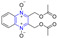	Antibacterial	[44]
Dioxidine	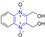	Antibacterial	[44]
NC-190	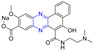	Anticancer	[45]
NC-182	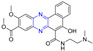	Anticancer	[46]
Erdafitinib	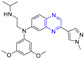	Anticancer	[47]
Pralatrexate	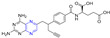	Anticancer	[48]
Methotrexate	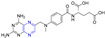	Anticancer	[49]
Selinexor	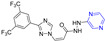	Anticancer	[50]
Gilteritinib	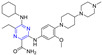	Anticancer	[51]
Grazoprevir	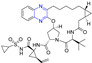	Anti-hepatitis C virus	[52]
Telaprevir	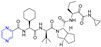	Anti-hepatitis C virus	[53]
Triamterene	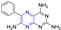	Potassium-sparing diuretics	[54]
Folic acid	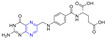	Reduction of the neural tube defect risk	[55]
Selexipag (NS-304)	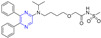	Pulmonary arterial hypertension	[56]
2-Pyrazinecarboxamide	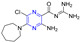	Antituberculosis	[57]
Thionazine	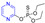	Insecticide and nematicide	[58]
Tetramethylpyrazine	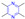	Anti-inflammatory	[59]
Sulfalen	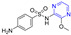	Urinary tract infection	[60]
Brimonidine	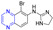	Glaucoma	[61]
Echinomycin	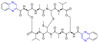	Antibacterial	[62]
Chloroquinoxaline sulfonamide	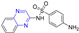	Anticancer	[63]
Brimondine	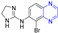	Rosacea	[64]
Pyrazine-2-diazohydroxide	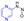	Antitumor	[65]
Acipimox	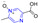	Hyperlipidaemia	[66]
Sulfalene (sulfamethoxypyrazine)	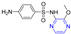	Resistant falciparum malaria and antibacterial	[67]
Mirfentanil	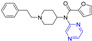	Selectivity for the µ opioid receptor; analgesic	[68]

## List of Abbreviations

HCV	Hepatitis C virus
RdRp	RNA-dependent RNA polymerase
SRB	Sulforhodamine B
HMEC-2	Human microvascular endothelial cell line
SH-SY5Y	Human neuroblastoma cell line
ChEs	Cholinesterase
ROS	reactive oxygen species
Cyt-c	Cytochrome-C
ADP	Adenosine diphosphate
PARP	Poly(ADP-ribose) polymerase
MCAO	Middle cerebral artery occlusion
HBV	Hepatitis B virus
Tph-1	Tryptophan hydroxylase 1
ADME	Absorption, distribution, metabolism, excretion
LPS	Lipopolysaccharide
NO	Nitric oxide
NAs	Nanoaggregates
DTT	Dithiothreitol
PPT	Podophyllotoxin
